# Digital interventions to moderate physical inactivity and/or nutrition in young people: a Cancer Prevention Europe overview of systematic reviews

**DOI:** 10.3389/fdgth.2023.1185586

**Published:** 2023-07-04

**Authors:** Kevin T. McDermott, Caro Noake, Robert Wolff, Linda Bauld, Carolina Espina, Jérôme Foucaud, Karen Steindorf, Mangesh A. Thorat, Matty P. Weijenberg, Joachim Schüz, Jos Kleijnen

**Affiliations:** ^1^ Kleijnen Systematic Reviews Ltd., York, United Kingdom; ^2^ Usher Institute and SPECTRUM Consortium, University of Edinburgh, Edinburgh, United Kingdom; ^3^ Environment and Lifestyle Epidemiology Branch, International Agency for Research on Cancer (IARC/WHO), Lyon, France; ^4^ Institut National du Cancer (INCa), Boulogne-Billancourt, France; ^5^ Université Sorbonne Paris Nord, Laboratoire Éducations et Pratiques de Santé (UR 3412), Bobigny, France; ^6^ Division of Physical Activity, Prevention and Cancer, German Cancer Research Center (DKFZ) and National Center for Tumor Diseases (NCT) Heidelberg, Heidelberg, Germany; ^7^ Breast Services, Guy's Hospital, Guy's and St Thomas’ NHS Foundation Trust, Great Maze Pond, London, United Kingdom; ^8^ Centre for Cancer Prevention, Wolfson Institute of Population Health, Queen Mary University of London, London, United Kingdom; ^9^ School of Cancer & Pharmaceutical Sciences, Faculty of Life Sciences & Medicine, King's College London, London, United Kingdom; ^10^ Department of Epidemiology, GROW School for Oncology and Reproduction, Maastricht University, Maastricht, Netherlands

**Keywords:** digital health, diet, physical activity, cancer, systematic reviews, public health, evidence synthesis, evidence appraisal

## Abstract

**Background:**

Strategies to increase physical activity (PA) and improve nutrition would contribute to substantial health benefits in the population, including reducing the risk of several types of cancers. The increasing accessibility of digital technologies mean that these tools could potentially facilitate the improvement of health behaviours among young people.

**Objective:**

We conducted a review of systematic reviews to assess the available evidence on digital interventions aimed at increasing physical activity and good nutrition in sub-populations of young people (school-aged children, college/university students, young adults only (over 18 years) and both adolescent and young adults (<25 years)).

**Methods:**

Searches for systematic reviews were conducted across relevant databases including KSR Evidence (www.ksrevidence.com), Cochrane Database of Systematic Reviews (CDSR) and Database of Abstracts of Reviews of Effects (DARE; CRD). Records were independently screened by title and abstract by two reviewers and those deemed eligible were obtained for full text screening. Risk of bias (RoB) was assessed with the Risk of Bias Assessment Tool for Systematic Reviews (ROBIS) tool. We employed a narrative analysis and developed evidence gap maps.

**Results:**

Twenty-four reviews were included with at least one for each sub-population and employing a range of digital interventions. The quality of evidence was limited with only one of the 24 of reviews overall judged as low RoB. Definitions of “digital intervention” greatly varied across systematic reviews with some reported interventions fitting into more than one category (i.e., an internet intervention could also be a mobile phone or computer intervention), however definitions as reported in the relevant reviews were used. No reviews reported cancer incidence or related outcomes. Available evidence was limited both by sub-population and type of intervention, but evidence was most pronounced in school-aged children. In school-aged children eHealth interventions, defined as school-based programmes delivered by the internet, computers, tablets, mobile technology, or tele-health methods, improved outcomes. Accelerometer-measured (Standardised Mean Difference [SMD] 0.33, 95% Confidence Interval [CI]: 0.05 to 0.61) and self-reported (SMD: 0.14, 95% CI: 0.05 to 0.23) PA increased, as did fruit and vegetable intake (SMD: 0.11, 95% CI: 0.03 to 0.19) (review rated as low RoB, minimal to considerable heterogeneity across results). No difference was reported for consumption of fat post-intervention (SMD: −0.06, 95% CI: −0.15 to 0.03) or sugar sweetened beverages(SSB) and snack consumption combined post-intervention (SMD: −0.02, 95% CI:–0.10 to 0.06),or at the follow up (studies reported 2 weeks to 36 months follow-up) after the intervention (SMD:–0.06, 95% CI: −0.15 to 0.03) (review rated low ROB, minimal to substantial heterogeneity across results). Smartphone based interventions utilising Short Messaging Service (SMS), app or combined approaches also improved PA measured using objective and subjective methods (SMD: 0.44, 95% CI: 0.11 to 0.77) when compared to controls, with increases in total PA [weighted mean difference (WMD) 32.35 min per day, 95% CI: 10.36 to 54.33] and in daily steps (WMD: 1,185, 95% CI: 303 to 2,068) (review rated as high RoB, moderate to substantial heterogeneity across results). For all results, interpretation has limitations in terms of RoB and presence of unexplained heterogeneity.

**Conclusions:**

This review of reviews has identified limited evidence that suggests some potential for digital interventions to increase PA and, to lesser extent, improve nutrition in school-aged children. However, effects can be small and based on less robust evidence. The body of evidence is characterised by a considerable level of heterogeneity, unclear/overlapping populations and intervention definitions, and a low methodological quality of systematic reviews. The heterogeneity across studies is further complicated when the age (older vs. more recent), interactivity (feedback/survey vs. no/less feedback/surveys), and accessibility (type of device) of the digital intervention is considered. This underscores the difficulty in synthesising evidence in a field with rapidly evolving technology and the resulting challenges in recommending the use of digital technology in public health. There is an urgent need for further research using contemporary technology and appropriate methods.

## Introduction

A healthy lifestyle with good nutrition and regular physical activity (PA) is known as a preserver of health and wellbeing (1) but many modern diseases including cancer (2) are related to poor lifestyle, inactivity and/or poor diet. Studies have shown that nearly 40% of cancer cases are related to known modifiable risk factors, and therefore preventable (3). These main risk factors include (but are not limited to) tobacco, an unhealthy diet, insufficient PA, being overweight, and alcohol consumption (4). A potential consequence of poor diet, and inactivity is becoming overweight and potentially developing obesity, and it is estimated that almost 4% of cancer cases worldwide are related to excessive bodyweight (5). A recent review reported that there is moderate to strong evidence to link excess bodyweight and obesity, and a sedentary lifestyle to multiple cancers (2).

It is imperative that the impact of lifestyle and long-term health outcomes continue to be studied and results disseminated to the public. The role of PA in protection from cancer has continued to receive considerable attention with new insight into the molecular mechanisms, and research on the signalling effects of adipokines and myokines becoming more prominent (6). However, despite unhealthy lifestyle choices generally being well known to increase risk of cancer, there is not a full and consistent understanding of this across society and knowledge of how this may translate into daily sensible choices is not so well developed (7).

Public health education on the risks of cancer as a consequence of poor diet, excess of bodyweight and inactivity is therefore important for two principal reasons, (1) That lifestyle changes can ultimately reduce risk of developing cancer in society, and, (2) To ensure that this knowledge is appropriately disseminated so that the public can understand how to make positive choices. By promoting risk awareness and encouraging health-conscious behaviour, individuals will be better educated about healthy lifestyle choices which are then arguably more likely to be made.

Digital health technologies which are defined by the World Health Organisation (WHO) as targeted client communication, untargeted client communication, client to client communication, personal health tracking and on-demand information services to clients could therefore provide an effective method to educate and inform about such risk factors. Devices which are programmable and widely used such as computers, mobile phones, IPADs etc can therefore be useful tools to facilitate interventions and healthcare advice which could potentially yield considerable benefits to public health. Such technology can help improve education, information delivery and risk awareness and facilitate health-conscious behaviour changes, particularly in young people and children, who are generally more technology aware throughout their formative years. One particular application of digital technologies in delivering healthcare services can be seen in the growth of Mobile Health (mHealth) initiatives to deliver public health interventions, which have increased, especially in younger populations, who utilise smartphones for much of their routine day to day life. This represents a behavioural trait that has coined the humorous term “phono-sapiens” (8, 9). mHealth platforms represent a particular method of digital intervention and healthcare delivery, can be malleable and user responsive which may provide opportunities for public health specialists to target many people and also monitor people's behaviour in “real-time” (10).

This review of systematic reviews aimed to synthesise the evidence for the effectiveness of digital interventions on consumption of unhealthy food/Sugar Sweetened Beverages(SSBs) and physical inactivity in adolescents and young people. Our objective was to ascertain (1) are digital interventions aimed at young people effective in addressing physical inactivity and poor dietary choices? and (2) What is the quality and strength of the systematic review evidence?.

## Methods

**Figure 1:**
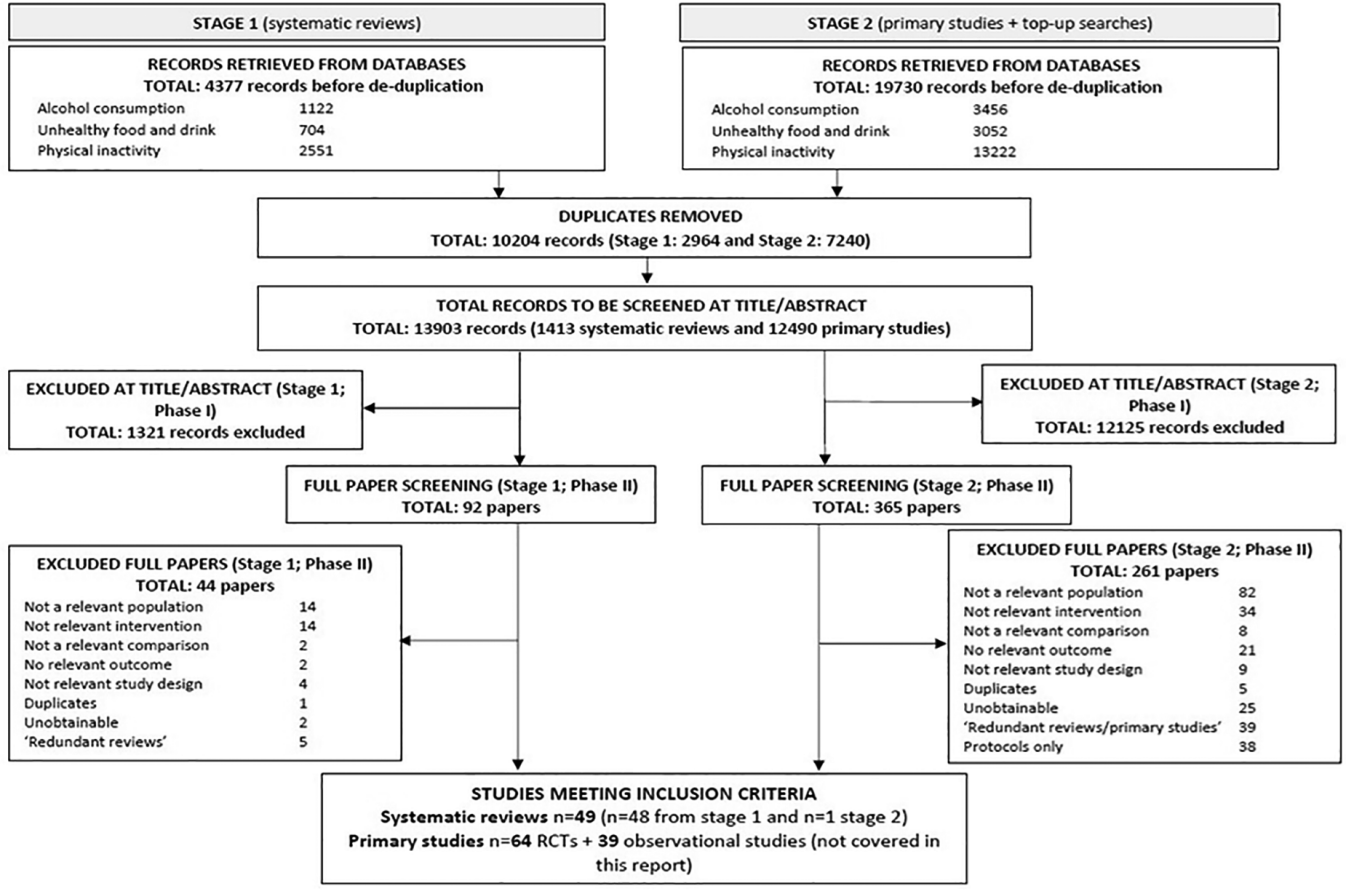
PRISMA flow chart. PRISMA flow chart detailing literature searches of the wider project (including the topics: unhealthy food and drink, alcohol consumption, and physical activity and inactivity). Systematic reviews relevant to diet and activity/inactivity discussed in this article represent 24 included systematic reviews.

It is important to clarify that this paper addressing the systematic review of the evidence for digital interventions and impact on consumption of unhealthy food/SSBs and physical (in)activity has emerged from a wider project investigating the impact of digital technologies on a variety of lifestyle factors. This review will report on evidence for physical (in)activity and nutrition only. For this reason however, search strategies (Supplementary Appendix S1), excluded studies (Supplementary Appendix S2) and specific numerical data in the Preferred Reporting Items for Systematic Reviews and Meta-Analyses (PRISMA) flow chart (Figure 1) are broader than the topics of diet and PA alone. Due to the large overlap between these topics and to ensure completeness all search results were imported into a single EndNote library and screened for all areas of interest. We also highlight that there may be sections of this manuscript with similar or identical text to that in another review of reviews which was derived from the same research project and conducted by the same research team (11).

### Eligibility criteria

Studies were selected for inclusion based on the following criteria:

• Population: Children, adolescents and young adults aged 10–24 years, including mean age within this range or a subgroup within this range. The age range of 10–24 years was selected as this represented the age range of full-time school and university level education, people in this age range were more routinely exposed to digital technology, and where behavioural and lifestyle patterns were being formed. Typically, we have generally considered children to be aged 10–16 years, adolescents to be 16–18 years, and young adults to be aged 18–24 years, however we emphasise that these are not absolute definitions and some studies may include participants that can overlap into more than one category. Subgroups: (school-aged children [includes adolescents; ≤ 18 years]; college/university students; young adults [≥19 years]; both adolescents and young adults [any age range <25 years]).
• Intervention: Digital interventions addressing the following risk factors for cancer: unhealthy food/SSBs and/or physical inactivity. The definition of digital interventions followed that of the WHO which includes: targeted client communication, untargeted client communication, client to client communication, personal health tracking and on-demand information services to clients (12). All interventions delivered by a healthcare or other professional or peer as well as those intended to be self-guided were included. A digital intervention was generally understood to be delivered primarily through programmable computer or mobile device (laptop, mobile phone, tablet, or smart watch). It should be noted that a device (computer, mobile phone, tablet etc), could be used to receive intervention *via* internet (email, apps, website login) or phone network connectivity (SMS, MMS) and these distinctions should be considered when reviewing the evidence presented here, i.e., digital or internet may be synonymous and interchangeable with mobile phone or computer. It is important to note that interventions could often fit into more than one category and the final classification and grouping in this article was based on reviewers' opinions and discussions.
• Comparators: Any comparators were eligible. This included studies where the control group received no intervention, is on a waiting list or received an active intervention (digital or non-digital such as printed or face-to-face).
• Outcomes: self-reported or objective measures related to reduction of physical inactivity or increase uptake of healthy foods. Reduction in cancer incidence because of the interventions (if available) was eligible. Relevant outcomes were those relating to quantity, frequency and intensity of unhealthy food and drink consumption and physical exercise. Adverse events (unintended consequences) relating to the interventions were also of interest.
• Systematic reviews were eligible. This included any study labelled by the study authors as a systematic review irrespective of quality.

### Literature search and screening

Each area of interest in the wider project, including diet and PA, was addressed with separate strategies, which were structured using search terms for general and question-specific digital interventions. The overall search strategy for the broader project was conducted in two stages. During stage 1, a rapid appraisal to identify existing systematic reviews and health technology assessments (HTA) was conducted.

The following databases and organisational websites were searched in April 2021 for relevant studies, from database inception to present (see Supplementary Appendix S1):

• KSR Evidence (www.ksrevidence.com).
• Cochrane Database of Systematic Reviews (CDSR) (Wiley).
• Database of Abstracts of Reviews of Effects (DARE) (CRD).
• Health Technology Assessment Database (HTA)(CRD).
• Epistemonikos (https://www.epistemonikos.org/).

Additionally manual searching of the following resources was conducted by reviewers to identify any relevant publications.

• World Cancer Research Fund (WCRF) (https://www.wcrf-uk.org/)
• International Agency for Research on Cancer (IARC) (https://www.iarc.fr/)
• World Health Organization (WHO) (https://www.who.int/health-topics/cancer)

Once the main relevant systematic reviews and HTA evidence were identified for each research question, a series of more focussed rapid review searches were carried out (stage 2). Appropriate date limits were defined in relation to each topic's systematic reviews evidence base (2015 for unhealthy food and drink and physical inactivity and 2016 for alcohol consumption). The following databases were searched for relevant studies:

• MEDLINE (Ovid)
• MEDLINE In-Process Citations, Daily Update & Epub Ahead of Print (Ovid)
• Embase (Ovid)

Search strategies were developed specifically for each database and the keywords adapted according to the configuration of each database. Due to the broad nature of the wider topic the review team recognised that the free text terms included in the strategies were not exhaustive, but the combination of the use of subject headings where available and the checking of reference lists in included studies was used to reduce the loss of recall. Searches were not limited by language or publication status (unpublished, published, in press, and in progress).

Titles and abstracts identified through electronic database and web searching were independently screened by two reviewers. Subsequently, full texts were independently examined in detail by two reviewers to determine whether they met the criteria for inclusion in the wider research project (see Supplementary Appendix S2 for studies excluded at this stage). Any discrepancies between reviewers were resolved through discussion or the participation of a 3rd reviewer. At this stage articles were categorised by the specific research question they addressed, in this case by diet and PA outcomes. The study selection process is detailed in accordance with the PRISMA statement (13).

### Data extraction

Data extraction was performed by teams of two reviewers. One reviewer extracted the data, and a second reviewer checked the extracted data against the original review. Any discrepancies were resolved through discussion with a third reviewer.

For systematic reviews, the data extraction comprised of basic information [author, year, years range and number of relevant primary studies, review type (diet/PA), intervention type, search end date, type of included study designs, best data available], information on population, intervention, comparator, and outcomes (PICO) and the overall conclusions.

### Risk of bias (RoB) assessment

The RoB was assessed using ROBIS (14). Two reviewers independently assessed study quality and any discrepancies were resolved through discussion and consensus or the intervention of a third reviewer. Where a review was deemed to contain inadequate methods, or insufficient reporting, to ensure confidence in bias limitation, the review was assessed as being low quality.

### Statistical analyses

A narrative summary of the studies is presented with a summary of the main study characteristics tabulated. No additional quantitative data synthesis was performed.

Emphasis was put on recent reviews, reviews of higher quality based on ROBIS scores and reviews where meta-analysis was conducted. Where reviews carried out a relevant meta-analysis, the pooled results were included. Conclusions from qualitative and/or older reviews were briefly summarised in narrative. Given the rapidly developing technology that exists, reviews were considered as possibly out-of-date if they had a latest search date before 2016 as they were unlikely to represent digital technology that is current, widely used, or advanced enough to have optimal interactivity and features. However, where other evidence was limited these older reviews were included and variously introduced.

The studies were categorised based on (1) the type of population as described in the paper or based on age provided in the paper (school-aged children [includes adolescents; ≤ 18 years]; college/university students; young adults [≥19 years]; both adolescents and young adults [any age range <25 years]) and (2) type of intervention [mobile phone; computer only; internet only; games; digital (any); other].

## Results

### Characteristics of included studies

The stage 1 systematic literature search for systematic reviews retrieved a total of 4,377 records, with 704 being relevant to unhealthy food/drink and 2,551 being relevant to physical (in)activity. The stage 2 systematic search identified 19,730 records, with 3,052 being relevant to unhealthy food/drink and 13,222 relevant for physical (in)activity. After de-duplication and screening, 49 systematic reviews were identified for the broader project area. Of these, 24 systematic reviews met the diet/PA relevant inclusion criteria.

24 reviews of unhealthy food and drink and physical inactivity were included (See Table 1). Across the reviews, these two topics tended to be considered together. Two of the reviews also considered alcohol consumption (31, 34). Relevant outcomes were those relating to quantity, frequency and intensity of unhealthy food consumption and PA; and any adverse events (unintended consequences) relating to the interventions. Reviews of unhealthy food and drink, and PA were firstly grouped by type of interventions and further by population.

**Table 1:** Characteristics of included systematic review.

Review	Type of synthesis	Summary of main aims	Inclusion/exclusion criteria	No of included studies*	Relevant main outcomes	RoB	Searches conducted up to**	Review overall conclusions	Relevant points (‘Take home’ summary)
**Mobile phone-based interventions**									
**Both adolescents and young adults**									
Direito 2017 (15)	Effect estimates	To compare the effectiveness of mHealth interventions to promote PA and reduce sedentary behaviour vs. a comparator exposed to usual care/minimal intervention.	*Design*: RCT's *Participants*: Free-living individuals (young people ≤18 years and adults ≥18 years) *Intervention*: Mobile interventions *Comparator*: Usual care or no intervention	4	PA	High	11th January 2015	Current mHealth interventions have a small effect on PA/sedentary behaviour.	Included 21 studies in their review but only four were clearly relevant to the age group of this project. No conclusions were drawn on this age group. searches were early 2015 and likely outdated. RoB was high.
Dute 2016 (16)	No results	To explore how mobile apps can contribute to the promotion of healthy nutrition, PA, and prevention of overweight in adolescents and students.	*Design*: Not reported *Participants*: Adolescents and students (age range 12 to 25 years old) *Intervention:* Mobile applications *Comparator:* Not reported	7	Not stated	High	November 2013	Despite large potential and abundant usage by young people, limited research is available on apps and health promotion for adolescents. Apps seem to be a promising health promotion strategy as a monitoring tool. Apps can enable users to set targets, enhance self-monitoring, and increase awareness. Three apps incorporated social features, making them “social media,” but hardly any evidence appeared available about their potential.	Limited research on apps for health promotion relevant to the 12 to 25 age group investigated. Only two of the 15 studies included in this review investigated the effect of using the apps. High RoB and outdated searches meaning up to date research likely missed.
**School-aged children**									
He 2021 (17)	Meta-analysis	To determine the effectiveness of smartphone-based interventions for improving PA in children and adolescents.	*Design*: RCTs *Participants*: Children and adolescents (age range 6 to 18 years) *Intervention:* Smartphone (text messaging and/or app) *Comparator:* Control groups not using smartphone technology	9	PA levels	High	June 29, 2020	The evidence of meta-analysis shows that smartphone-based intervention may be a promising strategy to increase total PA and steps in children and adolescents.	Smartphone intervention improved PA (SMD: 0.44, 95% CI: 0.11 to 0.77). Total PA, (WMD: 32.35 min, 95% CI: 10.36 to 54.33) and daily steps were improved (WMD: 1,185, 95% CI: 303 to 2,068), but no improvement was noted for moderate-to-vigorous PA (WMD: 3.91, 95% CI: −1.99 to 9.81, not clearly reported, assumed to also be minutes per day). Searches were recent (June 2020) but there was a high RoB.
Langarizadeh 2021 (9)	Qualitative	To appraise the potency of interventions based on mobile phone apps compared with other interventions to manage obesity and increase PA	*Design*: RCTs or quasi-experimental designs *Participants*: Healthy children and adolescents (18 years old and younger) *Intervention*: Mobile application *Comparator:* Usual care or traditional weight loss program	3	Weight-related (i.e., body mass index) and PA level measures	High	December 2020	The findings from these primary studies, although slightly mixed, provide support for further research with the implementation of mobile apps as an additional approach for combating childhood obesity.	Relatively recent searches but high RoB. Reports mixed results derived from small number of studies and no meta-analysis.
Ludwig 2018 (18)	Qualitative	To assess the effectiveness and use of theory of SMS interventions for improving physical activity and sedentary behaviour in youth	*Design*: RCTs or quasi-experimental *Participants*: Adolescents (between the ages of 10-19 years) with or without known morbidities *Intervention:* Text messages *via* a mobile phone ± mobile app/wearable trackers/online or school program *Comparator*: Usual care, another intervention, or no intervention	13	PA and sedentary behaviour (self-reported & objective)	High	November 8, 2017	Some studies in this review showed promising results for using SMS to improve PA and sedentary behaviour in youth.	Heterogeneous regarding study duration, participant characteristics, intervention content, and outcome measures. Findings were equivocal with some studies showing promise. RoB was high and there was no meta-analysis.
Villasana 2020 (19)	Qualitative	To review studies related to the promotion of healthy nutrition and physical activity habits in teenagers using mobile devices	*Design:* Not reported *Participants*: People aged 13 and 18 years old *Intervention*: Mobile interventions *Comparator:* Not reported	5	Dietary consumption or PA levels	High	Not reported	Review identified studies with different methodologies for the assessment of the acceptability of the use of mobile applications for the promotion of nutrition and physical activity habits with gamification.	Recent review but uncertain final search date. High RoB and no meta-analysis.
**Young adults only**									
Kim 2020 (8)	Meta-analysis	To identify the effects of smartphone-based health intervention programs provided for young adults on health outcomes, including PA promotion	*Design*: RCTs and quasi-experimental designs *Participants*: Young adults and adults (19 to 35 years) *Intervention*: Smartphone-based apps *Comparator:* Usual care or no comparator	2	Weight-related (i.e., body mass index), PA level measures and fruit/vegetable consumption	High	May 2019	The meta-analysis showed that smartphone-based health interventions significantly affect weight loss and increase PA. This study provides modest evidence for using smartphone health programs to improve young adults’ PA, weight control, and body mass index. Future research is needed.	Identified only five studies providing interventions to improve PA using smartphone applications for young adults. Not all studies were relevant to the age group of this project. For the whole population, the meta-analysis showed that smartphone-based health interventions generated increased PA (SMD: 2.59, 95% CI: 1.00 to 4.18 and weight loss WMD: −2.80 kg, 95% CI: −4.54 to −1.06 kg). Relatively up to date searches but had high RoB. Analysis was based on population with irrelevant age groups included.
**Game-based interventions**									
**Both adolescents and young people**									
Gao 2020 (20)	Qualitative	To synthesize studies carried out in field-based settings (e.g., homes, schools, and communities) and discern the effectiveness on body composition and objectively measured PA through rigorous, true experimental studies	*Design*: RCT's *Participants*: Children and/or adolescents (19 years and younger) *Intervention:* AVGs *Comparator:* Usual activities or no intervention	11	Weight-related (i.e., body mass index) and PA level measures	High	December 2019	AVGs are deemed a promising addition to promote PA and health among overweight and obese youth with the goal of fighting childhood obesity.	Used a variety of different game interventions. Rated at high RoB and no meta-analysis conducted. Results were mixed with some potential in those who are overweight.
Oliveira 2020 (21)	Meta-analysis	To investigate the effectiveness of AVGs performed out of school (ie, home-based and laboratory- based) compared with minimal intervention on obesity-related outcomes and PA levels in children and adolescents.	*Design*: RCT's and quasi RCT's. *Participants:* Children and adolescents (aged 2 to 19 years old) *Intervention*: AVGs *Comparator:* Usual care, no intervention, education, advice, inactive video games, and waiting list	7	Weight-related (i.e., body mass index), PA level measures	High	October 2018	AVGs were better than minimal intervention in reducing body mass index and body weight, but not for PA in young people.	AVGs were not more effective than the control group for increasing PA levels at the short-term (6 trials; SMD: 0.06; 95% CI:−0.19 to 0.31). AVGs were not more effective compared with minimal intervention at intermediate-term follow-up (4 trials; SMD: 0.22; 95% CI:−0.09 to 0.52). Evidence was stated to be moderate quality. Review had high RoB and not all included studies were relevant were to this age group.
**School-aged children**									
Lamboglia 2013 (22)	Qualitative	To evaluate the use of exergaming as a strategic tool for the promotion of healthy behaviours in the fight against childhood obesity.	*Design*: RCT, observational studies *Participants*: Children and adolescents (age 6 to 15 years old) *Intervention:* Video games *Comparator:* Not reported	6	Energy expenditure, PA levels, body measurements (e.g., weight)	High	April 2012	Exergaming was found to increase PA levels, energy expenditure, maximal oxygen uptake, heart rate, and percentage of PA engaged in and to reduce waist circumference and sedentary screen time.	Results suggest positive impacts, but review had high RoB. No meta-analysis was conducted and searches were dated April 2012 so likely outdated research. Included children out with relevant age range.
Norris 2016 (23)	Qualitative	To present current evidence on school-based AVGs and their relationship with health and PA outcomes including motor skills in children and youth aged 5 years and over.	*Design*: RCT's and observational studies *Participants*: Children and adolescents (age range 5 to 18 years old) of any health status *Intervention:* AVGs in school (i.e. within a lesson, during breaktime, or before or after the school day) *Comparator:* Physical education lessons or no comparator	11	PA levels, fitness levels, body measurements and composition	High	May 2015	Insufficient evidence to recommend AVGs as efficacious health interventions within schools. Higher quality AVG research utilizing RCT designs, larger sample sizes, and validated activity measurements beyond the school day is needed.	Relatively outdated searches and high RoB. No meta-analysis conducted, Higher quality research was recommended. Not prioritised for discussion.
Ramirez-Granizo 2020 (24)	Qualitative	To carry out a systematic review of scientific literature addressing the effect of PA practice and the use of exergames through longitudinal and experimental studies	*Design*: Any *Participants*: Children *Intervention*: Exergames *Comparator*: Not specified	1	Body mass index and self-reported measure of PA (PAQ-C)	High	February 2020	Cognitive performance and speed of reaction are benefited by the practice of physical activity using these devices. Most studies prove that, independently of the degree of PA and the long-term effect it can produce, improvements are produced in terms of motivation, health status and participation of students, especially through sports games where the motor demand is higher.	Relatively recent searches but poor search strategy may have missed studies. Most studies were outside the scope of this review so conclusions may not be applicable. There was high RoB and no meta-analysis.
**Computer-based interventions**									
**College/University students**									
Kroeze 2006 (25)	Qualitative	To systematically reviews the scientific literature on expert-driven computer-tailored physical activity and nutrition education	*Design*: RCT's *Participants*: College students (at least 18 years old) *Intervention*: Computer-tailored intervention aimed at PA or nutrition behaviours in primary prevention *Comparator*: Generic or no information	1	Fibre intake	High	September 2004	There was a significant effect on fibre consumption in favour of the tailored intervention group and no effect on food choice at medium term. Overall, there seems to be potential for the application of computer tailoring for promoting healthy diets, but more research is needed.	Only one study included in this review was in the relevant age group for this manuscript so it is unclear if conclusions are relevant to the age group of our review. Search dates were 2004 and so likely included outdated research, had high RoB.
**Internet-based interventions**									
**School-aged children**									
An 2009 (26)	Qualitative	To provide scientific evidence regarding the effectiveness of Web-based weight management programs and suggest directions for future research to optimize the Internet effectively for weight management for all children and youth.	*Design*: RCT's *Participants*: Children and adolescents who are overweight *Intervention*: Web-based weight management programs *Comparator*: Any comparator	3	Body mass index, dietary consumption, PA levels, weight change or body dissatisfaction	High	April 2009	The evidence suggests potential for Web-based behavioural change programs for weight management in overweight children and adolescents. Future research should emphasize rigorous methodological adequacies, develop theory-based standardized frameworks, investigate types of interventions appropriate for boys and girls in this age group, evaluate long-term effect of interventions, and examine cost as well as clinical effectiveness.	High RoB, outdated searches and no meta-analysis. Limited relevant studies included. Not prioritised for discussion.
**Both adults and young adults**									
Chau 2018 (27)	Qualitative	To review efficacy of interventions that utilize social media for improving nutrition among adolescents and young adults.	*Design*: RCT's and observational studies *Participants*: Adolescents (10 to 19 years old) ad/or young adults (18 to 25 years old) who are healthy or have a health challenge (i.e., being overweight, obese or have a chronic disease) *Intervention:* A social media website, application or homegrown technology that allows users to communicate or share information with peers *Comparator*: Any comparator	8	Weight change, dietary consumption, PA levels	High	September 2016	Social media is a promising feature for nutrition interventions for adolescents and young adults. A limited number of studies were identified that included social media. A majority of the identified studies had positive outcomes. Most studies utilized basic social media features, did not evaluate the efficacy of social media components, and did not differentiate between the efficacy of social media compared to other delivery mechanisms.	Included limited relevant research that was likely relatively outdated and contained participants outside the age range of this review. High RoB and no meta-analysis. Not prioritised for discussion.
McIntosh 2017 (28)	Qualitative	To assess the effectiveness of E-health interventions for increasing PA levels in young people.	*Design*: All study designs *Participants*: Adolescents, Young adults *Intervention:* Web-based interventions *Comparator:* Included but not specified	10	PA	High	July 2016	E-health interventions are a very successful way to increase PA.	80% of included studies reported increased PA after intervention but included range of study designs, conducted no meta-analysis, and had high RoB. Relatively outdated searches.
Wickham 2018 (29)	Qualitative	To: 1) systematically assess the literature to determine which adolescent food literacy programs incorporate technology; 2) identify how technology is used in these programs; and 3) examine dietary intake outcomes to determine the specific effectiveness of technology-driven components.	*Design*: RCT's and observational studies *Participants*: Adolescents (age range 12 to 19 years old) *Intervention*: Internet-based interventions that included food literacy concepts (planning and managing, selecting, preparing, and eating healthy foods) and technology component *Comparator*: Any comparator	8	Increased knowledge and improved dietary behaviours (e.g., consumption of fruit or vegetables)	High	Unclear	Studies included between two and four constructs of food literacy. All reported positive changes in food intake with five reporting significant positive pre- and post-intervention changes. Few technology-driven food literacy-related studies exist within the literature. Due to variation in program design, delivery, and evaluation it is difficult to tease out the effect of the technology component.	Seven studies used internet or web-based platforms to access program components and all RCTs incorporated game elements. Search dates were unclear, so it is possible that relevant studies were missed, and there is a high RoB.
**Young adults only**									
Maher 2014 (30)	Effect estimates	To systematically review the current level of evidence regarding the effectiveness of online social network health behaviour interventions to influence tobacco and alcohol consumption, dietary intake, PA, and sedentary behaviour.	*Design*: RCT's *Participants*: Young adults (regardless of health status) *Intervention*: An online intervention delivered either wholly or in part, using an online social network to deliver a health behaviour change intervention *Comparator:* Any comparator	2	Self-reported PA, objective weight	High	December 12, 2012	To date there is very modest evidence that interventions incorporating online social networks may be effective; however, this field of research is in its infancy. The effect sizes were generally small.	High RoB, older searches with likelihood of missed research, only two of the studies in this review were in the age group of interest.
**Any Digital-based intervention**									
**School aged children**									
Champion 2019 (31)	Meta-analysis	To systematically review eHealth school-based interventions targeting diet, PA and other lifestyle factors and identify intervention characteristics associated with effectiveness.	*Design*: RCT's *Participants*: Adolescents (age range 11 to 18 years) *Intervention:* School-based prevention programme that was universal (ie, delivered to all students regardless of their level of risk) and that targeted two or more of the following behaviours: alcohol use, smoking, diet, PA, sedentary behaviour (screen time and sitting), or sleep; and were primarily delivered *via* eHealth methods (eg, the internet, computers, tablets, mobile technology, or tele-health). *Comparator:* No intervention, education as usual, or an alternate evidence-based intervention not delivered *via* eHealth (e.g., face to face).	25	Alcohol consumption (prevalence), fruit intake, fruit and vegetable intake, fat intake, PA (objective & self-report)	Low	March 14, 2019	eHealth school-based interventions addressing multiple lifestyle risk behaviours can be effective in improving PA, screen time, and fruit and vegetable intake. However, effects were small and only evident immediately after the intervention.	Low RoB, relatively recent and included meta-analysis. Increased fruit and vegetable intake (SMD: 0.11, 95% CI: 0.03 to 0.19) and both accelerometer-measured (SMD: 0.33, 0.05 to 0.61) and self-reported (SMD: 0.14, 0.05 to 0.23) PA. Small and immediate effects. No difference was reported for fat (after intervention: SMD: −0.06, 95% CI: −0.15 to 0.03) and SSBs or snack consumption combined (after intervention: SMD: −0.02, 95% CI: −0.10 to 0.06; follow-up: SMD: −0.06, 95% CI: −0.15 to 0.03)
Chen 2014 (32)	Qualitative	To evaluate the existing literature reported on the effectiveness of technology-based interventions in preventing obesity in adolescents and to explore components of these interventions that are associated with significant body mass index (BMI) outcomes.	*Design*: RCT's and observational studies *Participants*: Adolescents (aged 12 to 18 years old) *Intervention:* Technology-based interventions (delivered *via* e.g. Internet or active video games) *Comparator:* Any comparator (e.g. general information)	12	PA and diet behaviour, body composition and measurements (e.g. weight)	High	January 2014	Studies showed some improvement in the levels of physical activity and increase in healthy diet behaviour, thus, supported the use of technology in reducing unhealthy weight in adolescents. However, interventions are likely to only have a short-term impact on weight management. Future research should assess the long-term impact of technology-based interventions and evaluate mediators and moderators for weight change in adolescents.	Relatively outdated searches, no meta-analysis and high RoB. Not prioritised for discussion
Hamel 2011 (33)	Effect estimates	To examine evidence regarding computer- or web-based interventions to increase preadolescent and adolescent PA	*Design*: RCT's, Observational studies *Participants*: Pre-and adolescents (8 to 18 years of age) *Intervention:* Computer or web-based interventions *Comparator:* Not specified	5	PA or a PA-related health change (e.g. change in body mass index, weight, percent body fat or waist circumference)	High	2010	Computer and web-based interventions can promote PA among preadolescents and adolescents, particularly in schools	High RoB, searches as of 2010 mean up to date research will be missed out and no meta-analysis conducted. Not prioritised for discussion.
Shingleton 2015 (34)	Qualitative	To describe and evaluate the methods and efficacy of technology-delivered motivational interviewing interventions (TAMIs), discuss the challenges and opportunities of TAMIs, and provide a framework for future research	*Design*: Not stated *Participants*: Not stated *Interventions:* TAMIs, though motivational interviewing solely by videoconference was excluded though motivational interviewing solely by videoconference was excluded *Comparator:* Not stated	5	Various outcomes across multiple heterogenous studies	High	February 27, 2015	Researchers have used a range of technologies to deliver TAMIs suggesting feasibility of these methods. However, there are limited data regarding their efficacy, and strategies to deliver relational components remain a challenge. Future research should better characterize the components of TAMIs, empirically test the efficacy of TAMIs with RCTs, and incorporate fidelity measures.	Outdated searches mean likely missed recent research. Had high RoB and no meta-analysis was conducted. Only one included study was in age group relevant to the purpose of this review. Not prioritised for discussion.

**Other interventions**									
**School-aged children**									
Bohm 2019 (35)	Qualitative	To examine the effectiveness of interventions that use tools of mHealth, respectively wearable activity trackers, to promote and change PA among children and/or adolescents (alcohol, illegal drugs) and that were assessed for efficacy.	*Design*: RCT's and observational studies *Participants*: Children and/or adolescents *Intervention:* mHealth tool (mobile phones, smartphones, tablets, apps, or other devices) or a wearable device *Comparator:* No treatment or intervention	6	PA (self-reported & objective)	High	June 2018	No clear recommendations can be derived. There is a clear need for future studies to develop PA interventions grounded on intervention mapping with a high methodological study design for specific target groups to achieve meaningful evidence.	Wide range of varied interventions, high RoB and no meta-analysis. Six studies had relevant age group, no evidence was found for the effect of mHealth tools or wearable activity trackers, on PA-related outcomes.
**Both adolescents and young adults**									
Ridgers 2016 (36)	Qualitative	To examine the effectiveness of wearable activity trackers as a tool for increasing children's and adolescents’ PA levels.	*Design*: Not reported *Participants*: Children and adolescents (between ages 5-19 years) *Intervention*: Wearable device (wearable activity tracker) *Comparator*: Not specified	2	PA [self-reported & objective (i.e., step count)], sedentary behaviour	High	August 2016	There is a paucity of research concerning the effectiveness and feasibility of wearable activity trackers as a tool for increasing children's and adolescents’ PA levels. There is some preliminary data to suggest these devices may have the potential to increase activity levels through self-monitoring and goal setting in the short term.	High RoB and included only two studies relevant to this age group, neither of which reported positive outcomes, however searches were conducted in 2016 so recent research will have been missed.

*This may represent either the total number of included studies in reviews, or the number of studies included in any particular sub group, sub population, or outcome of a review that could be described separately and was relevant to the aims of this review.

**Emphasis was put on recent reviews, reviews of higher quality based on ROBIS scores, and reviews where meta-analysis was conducted. Where reviews carried out a relevant meta-analysis, the pooled results were included. Conclusions from qualitative and/or older reviews were briefly summarised in narrative. Given the rapidly developing technology that exists, reviews were considered as possibly out-of-date if they had a latest search date before 2016 as they were unlikely to represent digital technology that is current, widely used, or advanced enough to have optimal interactivity and features. However, where other evidence was limited these older reviews were included and variously introduced.

AVG, Active Video Games; BMI, Body Mass Index; CI, Confidence Interval; Ehealth, Electronic Health; Kg, Kilograms; MD, Mean difference; mHealth, Mobile Health; OR, Odds Ratio; PA, Physical Activity; RCT, Randomised Controlled Trial; RoB, Risk of Bias; SMD, Standardised Mean Difference; SMS, Short Messaging Service; TAMI, Technology-delivered Motivational Interviewing Interventions; WHO, World Health Organization; WMD, Weighted mean Difference.

Seven reviews (8, 9, 15–19) focused on mobile technology, five (20–24) specifically had a game component, just one (25) was categorised as “computer”, five (26–30) used the internet for delivery and four (31–34) took a broad digital approach. Two reviews (35, 36) were categorised as “other”. Bohm and colleagues covered a range of interventions including wearable devices (35) and Ridgers and colleagues focused on wearable devices (36).

Within each intervention group, an attempt was made to identify the types of interventions that were found to be effective whilst being mindful of the quality of the reviews and their particular population, if appropriate, the emphasis was put on the more recent and higher quality reviews. However, all reviews but one (Champion et al.) in this section were rated at high RoB.

For study selections process see PRISMA flow chart in Figure 1.

### RoB assessment

Of the 24 identified systematic reviews included, only one was rated as having a low RoB (31) (see Supplementary Appendix S3, RoB assessment).

### Cancer incidence

No reviews reported any cancer related outcomes.

### Adverse events

No reviews reported any adverse events or outcomes related to safety.

### Mobile phone-based interventions

Seven reviews focused on mobile interventions. All were of low quality. Four reviews were in adolescents aged 18 or under (9, 17–19), one covered only participants aged over 18 (8), and two covered both adolescents and adults (15, 16).

Two reviews were out-of-date (searches ending more than five years ago (15, 16). Dute and colleagues identified few relevant studies research on apps for health promotion relevant to the 12 to 25 age group investigated. Only two of the 15 studies included in this review investigated the effect of using the apps. Without providing clear justification, the authors stated that it was impossible to perform a meta-analysis on effectiveness, but concluded that the apps were “promising” (16). Direito and colleagues included 21 studies in their review but only four were clearly concerned with young people, and most included studies consisting of adult participants so no specific conclusions were drawn on this age group. Overall, the authors concluded that mHealth interventions had a small effect on PA/sedentary behaviour (15).

Vilasana and colleagues did not state the latest search date, but the review included studies up until 2018 (19). Participants in this review were aged between 13 and 18. This systematic review identified studies with different methodologies for the assessment of the acceptability of the use of mobile applications for the promotion of nutrition and PA habits with gamification (19).

Of the more up-to-date studies (8, 9, 17, 18), two (9, 18) provided a qualitative synthesis. Langarizadeh and colleagues concluded that findings from primary studies, although slightly mixed, provided support for further research with the implementation of mobile apps as an additional approach for combating childhood obesity. However, few of the included studies assessed the role of apps independently from other aspects of an intervention (9). Ludwig and colleagues assessed the role of text messaging interventions for improvement in PA and sedentary behaviour in adolescents aged 10 to 19 (18). A total of 13 studies were included in the qualitative analysis and were heterogeneous regarding to study duration, participant characteristics, intervention content, and outcome measures. The authors stated that findings were “equivocal” with some studies showing promise but no overall conclusions could be determined. The authors advised that more rigorous studies are needed to explore the relationship between intervention effectiveness and specific intervention components such as content and delivery (18).

Two of the more up-to-date studies provided a meta-analysis (8, 17). Kim and colleagues identified only five studies providing interventions to improve PA using smartphone applications for young adults (8). Not all studies were relevant to the age group of this project. For the whole population, the meta-analysis showed that smartphone-based health interventions generated increased PA (SMD: 2.59, 95% CI: 1.00 to 4.18) and weight loss (WMD: −2.80 kg, 95% CI: −4.54 to −1.06 kg) (8). He and colleagues aimed to determine the effectiveness of smartphone-based interventions for improving PA in children and adolescents (17). A total of nine studies were included in this review, including four mobile app interventions, three SMS text messaging interventions, and two app + SMS text messaging interventions. Compared with the control group, the use of smartphone intervention improved PA (SMD: 0.44, 95% CI: 0.11 to 0.77) although moderate to substantial heterogeneity was observed. Total PA was improved (WMD: 32.35 min, 95% CI: 10.36 to 54.33) and daily steps were improved (WMD: 1,185, 95% CI: 303 to 2,068), but no improvement was noted for moderate-to-vigorous physical activity (WMD: 3.91, 95% CI: −1.99 to 9.81, not clearly reported, assumed to also be minutes per day) (17).

### Game-based interventions

Five reviews focused on game interventions. None were rated at low RoB. All studies were in adolescents or adolescents and young adults aged 19 or under comprising Lamboglia and colleagues (22) (age 6 to 15 years), Norris and colleagues (23) (age 5 to 18 years), Gao and colleagues (20) (age 19 years and younger), Oliveira and colleagues (21) (age 2 to 19) and Ramirez-Granizo and colleagues (24) (age 6 to 16 years).

Two reviews were out-of-date (searches ending more than five years ago) (22, 23). Lamboglia et al. concluded that exergaming (the combination of interactive video games and physical exercise) increased PA levels, energy expenditure, maximal oxygen uptake, heart rate, and percentage of PA engaged in and reduced waist circumference and sedentary screen time. However, this review had a number of methodological limitations and included studies of children outside the range of this project (22). Norris and colleagues investigated active video games (AVGs) delivered in school and concluded that there was insufficient evidence to recommend them. Higher quality research using Randomised Controlled Trials (RCTs), larger sample sizes, and validated activity measurements was recommended (23).

Of the more up-to-date reviews (20, 21, 24), two provided a qualitative synthesis. Ramirez-Granizo and colleagues had a poor search strategy and may not have identified all relevant studies on PA and use of AVGs (24). Most of their included studies were outside the scope of our review in terms of population or relevant outcome and so conclusions on improvements in motivation, health status and participation in sports may not be specific or relevant for our review (24). Gao and colleagues identified 18 RCTs of AVGs relating to adolescents and young adults’ body composition and PA (20). However not all were relevant to the age group of this project. Most studies utilised commercially available AVGs including Nintendo Wii, Gamercize Bike, Xbox Kinect, and Dance Dance Revolution among others. Overall findings were mixed with two of three studies in overweight and obese people giving positive results and one finding no difference between intervention and control groups. For healthy people, eight studies included PA outcomes, of which three had positive effects from an AVG and five observed no differences between intervention and control. The review authors concluded that AVGs were promising for the promotion of PA among overweight/obese but that their role for healthy adolescents and young adults was unclear (20).

The remaining review by Oliveira and colleagues included a meta-analysis (21). Once again, not all 18 studies in this review were relevant to the target population or research question of this review due to their inclusion of studies with participants ranging from 2 to 19 years of age, or the outcome. Bearing in mind these limitations, the authors found moderate-quality evidence that the AVGs were not more effective than the control group for increasing PA levels at the short-term follow-up (6 trials; SMD: 0.06, 95% CI: −0.19 to 0.31). There was moderate-quality evidence that AVGs were not more effective compared with minimal intervention at intermediate-term follow-up (4 trials; SMD: 0.22, 95% CI: −0.09 to 0.52) (21).

### Computer-based interventions

One review by Kroeze and colleagues, rated at high RoB, focused solely on computer-based interventions (25). Just one of the studies included in this review was in the relevant age group for this project and was comprised of college students. It is unclear if the authors' overall conclusions on the potential of computer-based interventions tailored to promoting healthy diets is relevant to the age group of our review (25).

### Internet-based interventions

Five reviews focused on internet interventions. None were rated at low RoB. Two reviews included predominantly adolescents (26, 29) of which one included just overweight participants (26). One review focused on young adults only (30) and two included both adolescents and young adults (27, 28). All were at high RoB and none conducted a relevant meta-analysis.

Two reviews were out-of-date (searches ending more than five years ago) (26, 30). An and colleagues concluded that the evidence from RCTs suggested the potential for web-based behavioural change programs for weight management in overweight children and adolescents. The authors recommended further research (26). Maher and colleagues concluded that there was very modest evidence that interventions incorporating online social networks may be effective and that effect sizes were generally small. However only two of the studies in this review were in the age group of interest (30). Both of these older reviews had a number of methodological limitations.

Wickham and colleagues did not state the latest search date but the review is from 2018 and includes studies as late as 2016 (29). The focus of the review was the role of technology in food literacy. Eight studies were included. Seven of the studies used internet or web-based platforms to access program components and all RCTs incorporated game elements. However not all studies were solely technology-based. All included studies reported positive changes in healthy food intake with five reporting positive pre to post-intervention changes. Due to variation in program design, delivery, and evaluation the authors stated that it was difficult to ascertain the effect of the technology component (29).

The two most recent reviews (27, 28) included both adolescents and young adults. Both included a range of study types. Neither conducted a meta-analysis. The focus of Chau and colleagues was on a social media website, application or homegrown technology that allows users to communicate or share information with peers (27). McIntosh and colleagues investigated web-based interventions (WBI) more generally (28). Approximately half of the included studies in the review by Chau included some participants who fell outside the age range of this project (27) but all of the included studies in the review by McIntosh were in the correct age range for this project (28). Chau and colleagues concluded that social media is a promising feature for nutrition interventions for adolescents and young adults. However, they identified that most studies used only basic social media features, did not evaluate the efficacy of social media components, and did not differentiate between the efficacy of social media compared to other delivery mechanisms (27). McIntosh and colleagues stated that E-health interventions were a very successful way to increase PA. Eight of the 10 included studies had increases in PA as a result of an E-health intervention. The authors stated that studies that did not use a theoretical principle to underpin their intervention did not achieve successful results. They suggested that more research was required to identify which theoretical principles are best to help design interventions and also to assess the length of intervention required for optimal results (28). These two reviews are both positive but it should be remembered that they both had methodological limitations and may include studies that utilise outdated technology or have been superceded (27).

### Any digital-based interventions

Four reviews focused on digital interventions for unhealthy eating and physical exercise. Two reviews included adolescents but were deemed to be out-of-date and of poor methodological quality (32, 33). The other two reviews were more recent (31, 34). The review by Shingleton and colleagues was deemed to be out-of-date, provided only a qualitative synthesis and was at high RoB (34). It only included one study in the relevant age group for diet/PA outcomes. The review by Champion and colleagues, assessed digital interventions solely in school-aged children (31). It was higher quality, more up-to-date and included a meta-analysis.

Champion 2019 and colleagues found that eHealth interventions delivered in school settings and addressing multiple lifestyle risk behaviours can be effective in improving PA and fruit and vegetable intake. However, they noted that effects were small and only evident immediately after the intervention: increased fruit and vegetable intake (SMD: 0.11, 95% CI: 0.03 to 0.19) and both accelerometer-measured (SMD: 0.33, 95% CI: 0.05 to 0.61) and self-reported (SMD: 0.14, 95% CI: 0.05 to 0.23) PA. No difference was reported for fat (after intervention: SMD: −0.06, 95% CI: −0.15 to 0.03) and SSBs or snack consumption combined (after intervention: SMD: −0.02, 95% CI: −0.10 to 0.06; follow-up: SMD: −0.06, 95% CI: −0.15 to 0.03). The authors stated that further high quality, research was needed to develop eHealth interventions that can modify multiple behaviours and sustain long-term effects (31).

### Other interventions

Two reviews focused on interventions we classified as “other” (35). Bohm and colleagues covered a range of interventions including wearable devices (35) and Ridgers and colleagues focused on wearable devices (36). Both reviews targeted children and adolescents. Both were rated at high RoB and neither included a meta-analysis.

The review by Ridgers and colleagues was relatively out-of-date and only included two studies of relevance to our review based on participant age (36). These two relevant studies together did not demonstrate any clear and consistent effect of the interventions. However one of the studies observed self-reported significant increases in PA along with generally positive feedback on the specific intervention. However, the review as a whole included studies irrelevant to our review, reported generally non-significant differences and suggested the need for further research.

The review by Bohm and colleagues covered a range of mobile interventions (mobile phones, smartphones, tablets, apps or other devices) or a wearable device (35). Six of seven studies were relevant to our project in terms of participant age. Mobile health intervention delivery ranged from four weeks to 12 months, mainly using smartphone apps. The relevant study of wearable activity trackers covered a period of eight weeks. No evidence was found for the effect of mHealth tools or wearable activity trackers, on PA-related outcomes. The authors advised that further, higher quality studies were needed (35).

## Discussion

This review of systematic reviews aimed to synthesise the evidence for the effectiveness of digital interventions on consumption of unhealthy food/SSBs and physical inactivity in adolescents and young people.

No evidence was identified to suggest any impact on cancer incidence. This was consistent with the findings of our review on digital interventions and alcohol consumption (11). However this is not surprising given that cancer incidence in a younger population is generally lower and the population would need to be followed over a considerable period of time.

Findings related to PA participation and dietary intake suggest that despite the widespread use of digital technology and the primary research which has examined its effects, there is a paucity of robust and reliable systematic review evidence to support its use in raising awareness and for delivering public health recommendation in young people for the moderation of activity levels and dietary choices. Issues that seem apparent can be summarised into two categories, namely (1) What is the effectiveness of such interventions (do the results show that such interventions are effective?), and (2) quality and methodological limitations, (Is there sufficient quality and consistency in the data/methods for effects to be reliable?).

Limited systematic review evidence existed, as only 24 reviews were identified that fulfilled our criteria. However, the definitions of population and intervention that were used by each review, meant that results could not be easily grouped or considered together as they were often comparing different interventions, with different methods on different groups of people. At least one systematic review was included for each population type. Computer or other interventions were under-represented in the systematic reviews of unhealthy food and drink, and physical inactivity (<3 reviews across all populations). The majority of reviews included were low-quality with only 4.1% (1/24) of reviews judged as low RoB. Therefore, in commenting on our findings, it must be emphasised once again that unclear and overlapping definitions should be considered in the interpretation of results, with some interventions fitting into more than one category, and the final definitions being based on reviewers' opinions and discussions. The outcomes used in this review of reviews are those that were chosen in the included systematic reviews, that in turn had had to deal with the various definitions in the primary studies. This limited our options to use a comprehensive, clearly defined, and consistent set of outcomes and thereby nullified any opportunity for full systematic groupings and further meta-analysis.

For mobile interventions, games, computer interventions, internet or other interventions no firm conclusions can be drawn due to a high RoB of included systematic reviews. Across those reviews, three (two in mobile interventions and one in games) performed a meta-analysis. In two (one each in mobile and games), not all studies included in meta-analyses were eligible for this report (8, 21). For the second systematic review of mobile interventions, the use of the variety of smartphone interventions improved some aspects of children's and adolescents' PA (17). However, a high level of heterogeneity in the results across studies was found. The overall results should be interpreted with caution.

The highest quality evidence describing digital interventions was for school-aged children (with one systematic review with low RoB) with the results of a meta-analysis (31). The other three reviews were out-of-date. The review and meta-analysis by Champion and colleagues suggested that there is a small and short-term effect of eHealth school-based interventions delivered in the school environment and addressing multiple lifestyle risk behaviours in improving measures of PA, and fruit and vegetable intake but not for fat, SSBs or snacks.

## Strengths and limitations

Our review was developed using evidence from systematic reviews. Its strengths include comprehensive literature searches without language restriction and across a range of databases and resources, and the inclusion of the highest certainty evidence.

Several problems were identified with the eligible systematic reviews. Several reviews were deemed to be out-of-date, which is highly problematic in a rapidly changing technology field. Most reviews were at high RoB suggesting that their results and conclusions may not be reliable. In addition, many reviews included digital interventions that were defined in various ways by the authors. There was a high heterogeneity across the reviews in terms of populations, duration of interventions, content and personalisation, comparators, and outcomes, with additional heterogeneity across studies both in terms of methodology and results within the individual systematic reviews. Relatively up-to-date and good quality reviews were scarce.

While we conducted this process with rigour, there is always the potential that relevant evidence was missed. We acknowledge that our own searches were conducted in 2021 and consequently there may be systematic reviews published up to May 2023 that have therefore not been included in this overview. Digital technology, particularly smartphone related technology evolves rapidly with new apps and features, and new primary research may also have been published in this time that has trialled new intervention methods. However, we would suggest that we would not expect to see many systematic reviews recently published that meet our eligibility criteria. We would also expect to see any new reviews that did meet our criteria reporting on primary studies which are also within the reviews we have included. Finally, we consider it reasonable to suggest that any missed systematic reviews, with or without recent primary research included within, are unlikely to have major impact on the general findings and observations of this review.

**Figure 2:**
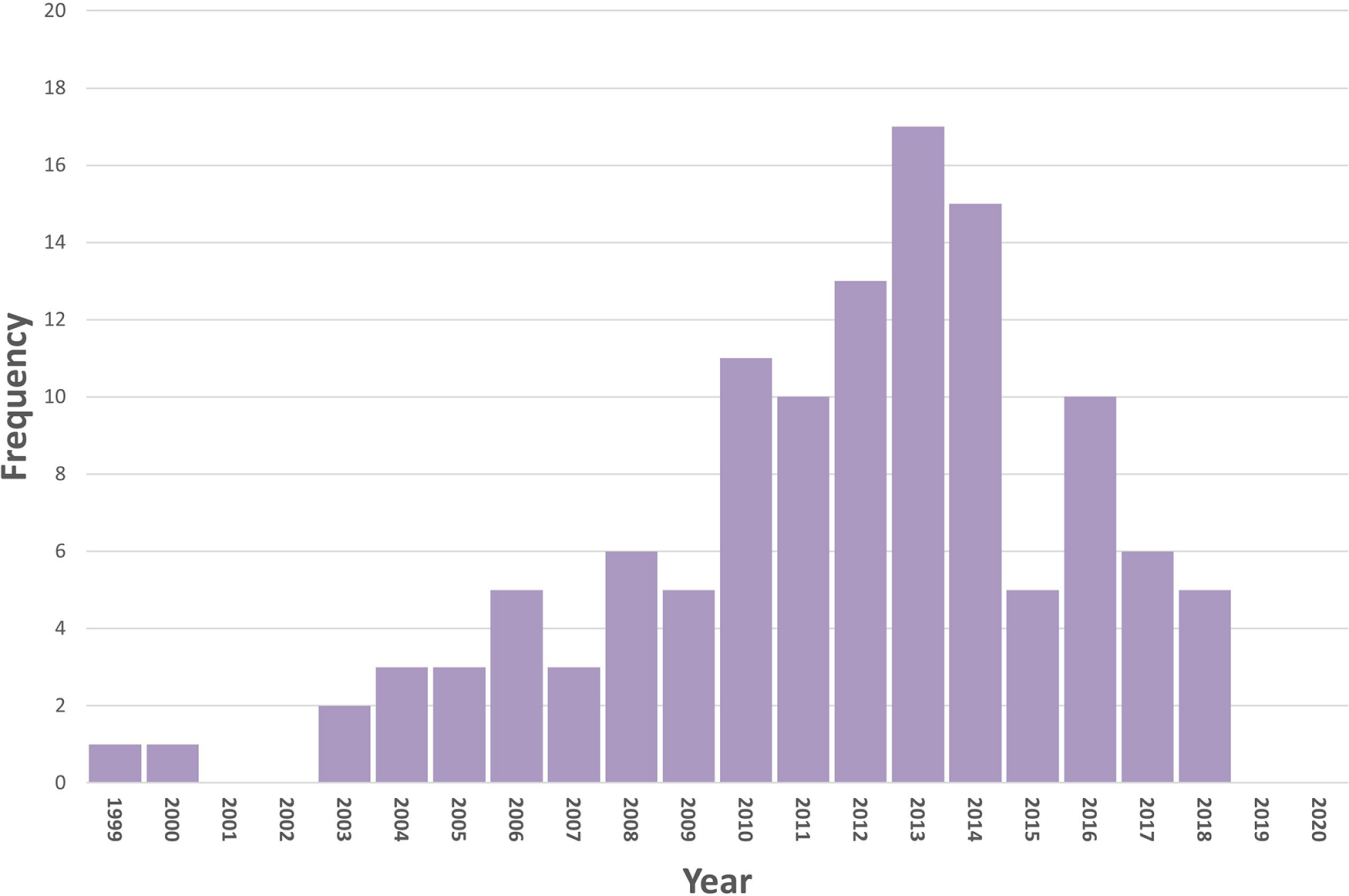
PRISMA flow chart. Primary studies identified by the systematic reviews of digital interventions to reduce unhealthy eating and physical inactivity. Numbers decline from 2013, emphasising that any recent literature had not been rigorously reviewed.

The review highlights a decline in numbers of primary studies included in the systematic reviews since 2014, emphasising that any recent literature had not been rigorously reviewed. As can be seen in Figure 2, primary studies included in the systematic reviews reach a peak in 2014. Interestingly a similar peak was observed in the primary studies of our review on digital interventions and alcohol (11). Older reviews will not include more recently published primary studies and will therefore not reflect more recent findings. Reviews varied in the inclusion criteria and the numbers of included studies. Thus, there is no certainty that all relevant studies were captured by included systematic reviews and so it is feasible that there may be relevant primary research that has not been identified here.

## Conclusions

This review aimed to survey the existing systematic review literature and assess the body of evidence. There is currently insufficiency of systematic review evidence to recommend the use of any digital interventions to reduce unhealthy food and drink consumption, and physical inactivity in adolescents/young adults. While there is insufficient evidence to form the basis of any recommendation to use digital interventions to improve public health, the available evidence at least suggests a need to conduct more research on this subject, particularly when concerned with eHealth interventions to moderate lifestyle choices in children in the school-based environment.

Future research is necessary that takes a more specific approach, to also address what may be more relevant variables in moderating the impact of an effect, such as feedback vs. non feedback. This is an important point, as definitions such as “computer”, “mobile phone” or “digital” are broad and generic and within them are a range of specific “treatments” delivered with specific protocols. Given the rapid evolution of digital technology and the wide variability within interventions, these future efforts may be helpful to elucidate the optimal digital strategy. While this was beyond the scope of this review, the risks of poor diet, excess bodyweight and physical inactivity underlines the need for a high impact digital strategy. One that is based on robust, reliable and specific evidence.

## Data Availability

The original contributions presented in the study are included in the article/Supplementary Material, further inquiries can be directed to the corresponding author.
