# Supplementary material for: Digital interventions to moderate physical inactivity and/or nutrition in young people: a Cancer Prevention Europe overview of systematic reviews
Source: Front Digit Health. 2023 Jul 4;5:1185586. doi: 10.3389/fdgth.2023.1185586 (PMC10393256; doi:10.3389/fdgth.2023.1185586)
Supplement: Supplementary file 1 [file Datasheet1.docx]

**Supplementary file 1. Search strategy**

As stated in the article this project was part of a wider review, during which searches were conducted in two stages, following a methodical approach:

Stage 1: Rapid appraisal to identify existing systematic reviews and health technology assessments (HTA) relevant to the broader project areas.

Stage 2: Rapid review searches: Series of targeted rapid review searches incorporating appropriate date limits defined in relation to each topic's SR evidence base, designed to identify any primary evidence or additional reviews that may be relevant.

In order to account for overlap amongst topics and to ensure completeness, results from each of the three search questions (unhealthy food and drink, alcohol consumption, and physical activity and inactivity) were combined in a single endnote library and screened for all areas of interest

**Stage 1: Rapid appraisal searches**

**Digital interventions for** **alcohol consumption**

| **Database** | **Dates** | **Results** |
| --- | --- | --- |
| KSR Evidence | up to 2021/04/27 | 112 |
| CDSR | up to 2021/04/Iss4 | 32 |
| DARE | up to 2015/03/31 | 44 |
| HTA | up to 2018/03/31 | 3 |
| Epistemonikos | up to 2021/04/27 | 931 |
| **Total** | | **1122** |

**KSR Evidence (www.ksrevidence.com): up to 2021/04/27**

**Searched 27.4.21**

1 (Internet or "World wide web" or app or apps or application or smartphone* or phone* or text messag* or SMS or web-based or interactive or video or youtube or whatsapp or facebook or social media or Instagram or Email* or Game* or Gaming or subscription or twitter or tweet* or snapchat) in All text 10919 results

2 (online NEAR (counsel* or coach* or diar*)) in All text 18 results

3 ((remote* or online or digital*) NEAR (deliver* or element* or program* or schedul* or advisor* or group* or participa* or tracker*)) in All text 408 results

4 e-coach* or zoom in All text 5 results

5 ((digital* or online* or remote* or tech) NEAR (application* or solution*)) in All text 91 results

6 (smart watch* or smartwatch* or wearable* or fittech or software or iphone or mobile device* or vlog* or vlogger* or influencer* or Phone track* or i-phone or android) in All text 9052 results

7 (Virtual reality NEAR (game* or active* or interface* or system* or simulat* or device* or display* or exercis*)) in All text 243 results

8 (VR NEAR (game* or active* or interface* or system* or simulat* or device* or display* or exercis*)) in All text 83 results

9 #1 or #2 or #3 or #4 or #5 or #6 or #7 or #8 in All text 19313 results

10 ("Generation Z" or "Gen Z" or "Young person" or "young people" or "Younger generation" or "School age" or "School child" or "school children" or "young adult" or " young adults" or "School aged" or school or schools) in All text 12255 results

11 (teen or teens or Teenager* or schoolage* or schoolchild* or freshman or freshmen or sophomore* or Student* or pupil* or adolescen* or Millennial* or "High school" or "Middle school") in All text 10214 results

12 (College* or Universit* or Youth or youths or Boy* or Girl* or "Young man" or "Young men" or "Young woman" or "Young women") in All text 26239 results

13 in All text 0 results

14 #10 or #11 or #12 in All text 35611 results

15 (alcohol or beer or beers or spirits or liquor or wine or "binge drinking" or "problem drinker" or "binge drinker" or "problem drinking") in All text 2853 results

16 ((problem* or excessive* or excess) NEAR (alcohol or drink*)) in All text 266 results

17 #16 or #15 in All text 2863 results

**18 #17 and #14 and #9 in All text 112 results**

**Cochrane Database of Systematic Reviews (CDSR) (Wiley): up to 2021/04/Iss4**

**Searched 22.4.21**

#1 MeSH descriptor: [Accelerometry] this term only 485

#2 MeSH descriptor: [Fitness Trackers] this term only 104

#3 MeSH descriptor: [Wearable Electronic Devices] explode all trees 459

#4 MeSH descriptor: [Wireless Technology] this term only 43

#5 MeSH descriptor: [Telemedicine] explode all trees and with qualifier(s): [instrumentation - IS] 197

#6 MeSH descriptor: [Telemedicine] this term only and with qualifier(s): [methods - MT] 1228

#7 MeSH descriptor: [Mobile Applications] explode all trees 748

#8 MeSH descriptor: [Internet] explode all trees 4171

#9 MeSH descriptor: [Smartphone] explode all trees 369

#10 MeSH descriptor: [Wearable Electronic Devices] this term only 87

#11 MeSH descriptor: [Fitness Trackers] this term only 104

#12 MeSH descriptor: [Electronic Mail] this term only 333

#13 MeSH descriptor: [Video Recording] this term only 1441

#14 MeSH descriptor: [Video Games] explode all trees 707

#15 MeSH descriptor: [Virtual Reality Exposure Therapy] this term only 195

#16 (Internet or "World wide web" or app or apps or application or smartphone* or phone* or text messag* or SMS or web-based or interactive or video or youtube or whatsapp or facebook or social media or Instagram or Email* or Game* or Gaming or subscription or twitter or tweet* or snapchat):ti,ab 111337

#17 (online near/3 (counsel* or coach* or diar*)):ti,ab 363

#18 ((remote* or online or digital*) near/3 (deliver* or element* or program* or schedul* or advisor* or group* or participa* or tracker*)):ti,ab 5128

#19 e-coach*:ti,ab 96

#20 ((digital* or online* or remote* or tech) near/3 (application* or solution*)):ti,ab 386

#21 (smart watch* or smartwatch* or wearable* or fittech or software or iphone or mobile device* or vlog* or vlogger* or influencer* or Phone track* or i-phone or android):ti,ab 18935

#22 (Virtual reality near/3 (game* or active* or interface* or system* or simulat* or device* or display* or exercis*)):ti,ab 1065

#23 (VR near/3 (game* or active* or interface* or system* or simulat* or device* or display* or exercis*)):ti,ab 599

#24 zoom:ti,ab 235

#25 #1 or #2 or #3 or #4 or #5 or #6 or #7 or #8 or #9 or #10 or #11 or #12 or #13 or #14 or #15 or #16 or #17 or #18 or #19 or #20 or #21 or #22 or #23 or #24 130078

#26 MeSH descriptor: [Adolescent] explode all trees 105120

#27 MeSH descriptor: [Adult] this term only 335465

#28 MeSH descriptor: [Young Adult] explode all trees 66544

#29 MeSH descriptor: [Child] explode all trees 56872

#30 MeSH descriptor: [Students] explode all trees 4505

#31 MeSH descriptor: [Schools] this term only 2050

#32 MeSH descriptor: [undefined] explode all trees 0

#33 ("Generation Z" or "Gen Z" or "Young person" or "young people" or "Younger generation" or "School age" or "School child" or "school children" or "young adult" or " young adults" or "School aged" or school or schools):ti,ab 36520

#34 (teen or teens or Teenager* or schoolage* or schoolchild* or freshman or freshmen or sophomore* or Student* or pupil* or adolescen* or Millennial* or "High school" or "Middle school"):ti,ab 64450

#35 (College* or Universit* or Youth or youths or Boy* or Girl* or "Young man" or "Young men" or "Young woman" or "Young women"):ti,ab 85241

#36 #26 or #27 or #28 or #29 or #30 or #31 or #32 or #33 or #34 or #35 491732

#37 MeSH descriptor: [Alcoholic Beverages] explode all trees 525

#38 MeSH descriptor: [Alcohol Drinking] explode all trees 3938

#39 MeSH descriptor: [Drinking Behavior] this term only 133

#40 (alcohol or beer or beers or spirits or liquor or wine or "binge drinking" or "problem drinker" or "binge drinker" or "problem drinking"):ti,ab 25248

#41 ((problem* or excessive* or excess) near/3 (alcohol or drink*)):ti,ab 2314

#42 #37 or #38 or #39 or #40 or #41 25847

**#43 #25 and #36 and #42 in Cochrane Reviews, Cochrane Protocols 32**

**Database of Abstracts of Reviews of Effects (DARE) (CRD): up to 2015/03/31**

**Health Technology Assessment Database (HTA) (CRD): up to 2018/03/31**

**Searched 27.4.21**

[**https://www.crd.york.ac.uk/CRDWeb/**](https://www.crd.york.ac.uk/CRDWeb/)

1 MeSH DESCRIPTOR Accelerometry EXPLODE ALL TREES 10

2 MeSH DESCRIPTOR Fitness Trackers EXPLODE ALL TREES 0

3 MeSH DESCRIPTOR Wearable Electronic Devices EXPLODE ALL TREES 66

4 MeSH DESCRIPTOR Wireless Technology EXPLODE ALL TREES 7

5 MeSH DESCRIPTOR Telemedicine EXPLODE ALL TREES WITH QUALIFIER IS 15

6 MeSH DESCRIPTOR Telemedicine EXPLODE ALL TREES WITH QUALIFIER MT 103

7 MeSH DESCRIPTOR Mobile Applications EXPLODE ALL TREES 5

8 MeSH DESCRIPTOR Internet EXPLODE ALL TREES 257

9 MeSH DESCRIPTOR Smartphone EXPLODE ALL TREES 0

10 MeSH DESCRIPTOR Wearable Electronic Devices EXPLODE ALL TREES 66

11 MeSH DESCRIPTOR Fitness Trackers EXPLODE ALL TREES 0

12 MeSH DESCRIPTOR Electronic Mail EXPLODE ALL TREES 12

13 MeSH DESCRIPTOR Telemedicine EXPLODE ALL TREES 423

14 MeSH DESCRIPTOR Video Games EXPLODE ALL TREES 34

15 MeSH DESCRIPTOR Virtual Reality Exposure Therapy EXPLODE ALL TREES 7

16 ((Internet or "World wide web" or app or apps or application or smartphone* or phone* or text messag* or SMS or web-based or interactive or video or youtube or whatsapp or facebook or social media or Instagram or Email* or Game* or Gaming or subscription or twitter or tweet* or snapchat)) OR ((online NEAR (counsel* or coach* or diar*))) OR (((remote* or online or digital*) NEAR (deliver* or element* or program* or schedul* or advisor* or group* or participa* or tracker*))) 4113

17 (e-coach* or zoom) OR (((digital* or online* or remote* or tech) NEAR (application* or solution*))) OR ((smart watch* or smartwatch* or wearable* or fittech or software or iphone or mobile device* or vlog* or vlogger* or influencer* or Phone track* or i-phone or android)) 800

18 ((Virtual reality NEAR (game* or active* or interface* or system* or simulat* or device* or display* or exercis*))) OR ((VR NEAR (game* or active* or interface* or system* or simulat* or device* or display* or exercis*))) 21

19 #1 OR #2 OR #3 OR #4 OR #5 OR #6 OR #7 OR #8 OR #9 OR #10 OR #11 OR #12 OR #13 OR #14 OR #15 OR #16 OR #17 OR #18 5153

20 MeSH DESCRIPTOR Adolescent EXPLODE ALL TREES 4594

21 MeSH DESCRIPTOR Adult 11018

22 MeSH DESCRIPTOR Young Adult EXPLODE ALL TREES 1941

23 MeSH DESCRIPTOR Child EXPLODE ALL TREES 4935

24 MeSH DESCRIPTOR Students EXPLODE ALL TREES 88

25 MeSH DESCRIPTOR Schools EXPLODE ALL TREES 200

26 MeSH DESCRIPTOR Universities EXPLODE ALL TREES 25

27 (("Generation Z" or "Gen Z" or "Young person" or "young people" or "Younger generation" or "School age" or "School child" or "school children" or "young adult" or " young adults" or "School aged" or school or schools)) OR ((teen or teens or Teenager* or schoolage* or schoolchild* or freshman or freshmen or sophomore* or Student* or pupil* or adolescen* or Millennial* or "High school" or "Middle school")) OR ((College* or Universit* or Youth or youths or Boy* or Girl* or "Young man" or "Young men" or "Young woman" or "Young women")) 11581

28 #20 OR #21 OR #22 OR #23 OR #24 OR #25 OR #26 OR #27 19997

29 MeSH DESCRIPTOR Alcoholic Beverages EXPLODE ALL TREES 8

30 MeSH DESCRIPTOR Alcohol Drinking EXPLODE ALL TREES 163

31 MeSH DESCRIPTOR Drinking Behavior EXPLODE ALL TREES 169

32 ((alcohol or beer or beers or spirits or liquor or wine or "binge drinking" or "problem drinker" or "binge drinker" or "problem drinking")) OR (((problem* or excessive* or excess) NEAR (alcohol or drink*))) 1053

33 #29 OR #30 OR #31 OR #32 1057

**34 (#19 and #28 and #33) IN DARE 44**

**35 (#19 and #28 and #33) IN HTA 3**

**Epistemonikos (Internet): up to 2021/04/27**

**Searched 27.4.21**

[**https://www.epistemonikos.org/en/advanced_search**](https://www.epistemonikos.org/en/advanced_search)

**Searched Title/Abstract fields:**

1) (title:((Internet or "World wide web" or app or apps or application or smartphone* or phone* or text messag* or SMS or web-based or interactive or video or youtube or whatsapp or facebook or social media or Instagram or Email* or Game* or Gaming or subscription or twitter or tweet* or snapchat) OR (online AND (counsel* or coach* or diar*)) OR ((remote* or online or digital*) AND (deliver* or element* or program* or schedul* or advisor* or group* or participa* or tracker*)) OR e-coach* or zoom OR ((digital* or online* or remote* or tech) AND (application* or solution*)) OR (smart watch* or smartwatch* or wearable* or fittech or software or iphone or mobile device* or vlog* or vlogger* or influencer* or Phone track* or i-phone or android) OR (Virtual reality AND (game* or active* or interface* or system* or simulat* or device* or display* or exercis*)) OR (VR AND (game* or active* or interface* or system* or simulat* or device* or display* or exercis*))) OR abstract:((Internet or "World wide web" or app or apps or application or smartphone* or phone* or text messag* or SMS or web-based or interactive or video or youtube or whatsapp or facebook or social media or Instagram or Email* or Game* or Gaming or subscription or twitter or tweet* or snapchat) OR (online AND (counsel* or coach* or diar*)) OR ((remote* or online or digital*) AND (deliver* or element* or program* or schedul* or advisor* or group* or participa* or tracker*)) OR e-coach* or zoom OR ((digital* or online* or remote* or tech) AND (application* or solution*)) OR (smart watch* or smartwatch* or wearable* or fittech or software or iphone or mobile device* or vlog* or vlogger* or influencer* or Phone track* or i-phone or android) OR (Virtual reality AND (game* or active* or interface* or system* or simulat* or device* or display* or exercis*)) OR (VR AND (game* or active* or interface* or system* or simulat* or device* or display* or exercis*))))

+

2) AND (title:(("Generation Z" or "Gen Z" or "Young person" or "young people" or "Younger generation" or "School age" or "School child" or "school children" or "young adult" or " young adults" or "School aged" or school or schools) OR (teen or teens or Teenager* or schoolage* or schoolchild* or freshman or freshmen or sophomore* or Student* or pupil* or adolescen* or Millennial* or "High school" or "Middle school") OR (College* or Universit* or Youth or youths or Boy* or Girl* or "Young man" or "Young men" or "Young woman" or "Young women")) OR abstract:(("Generation Z" or "Gen Z" or "Young person" or "young people" or "Younger generation" or "School age" or "School child" or "school children" or "young adult" or " young adults" or "School aged" or school or schools) OR (teen or teens or Teenager* or schoolage* or schoolchild* or freshman or freshmen or sophomore* or Student* or pupil* or adolescen* or Millennial* or "High school" or "Middle school") OR (College* or Universit* or Youth or youths or Boy* or Girl* or "Young man" or "Young men" or "Young woman" or "Young women")))

+

3) AND (title:((alcohol or beer or beers or spirits or liquor or wine or "binge drinking" or "problem drinker" or "binge drinker" or "problem drinking") OR ((problem* or excessive* or excess) AND (alcohol or drink*))) OR abstract:((alcohol or beer or beers or spirits or liquor or wine or "binge drinking" or "problem drinker" or "binge drinker" or "problem drinking") OR ((problem* or excessive* or excess) AND (alcohol or drink*))))

**Total = 931**

SRs = 281

Broad syntheses = 16

Structured summaries = 5

Primary studies = 629

**Digital interventions for Unhealthy Diet**

| **Database** | **Dates** | **Results Unhealthy diet** | **Results**  **SSBs** |
| --- | --- | --- | --- |
| KSR Evidence | up to 2021/04/29 | 33 | 23 |
| CDSR | up to 2021/04/Iss4 | 15 | 80 |
| DARE | up to 2015/03/31 | 12 | 2 |
| HTA | up to 2018/03/31 | 1 | 3 |
| Epistemonikos | up to 2021/04/27 | 276 | 259 |
| **Total** | | **337** | **367** |

**KSR Evidence (www.ksrevidence.com): up to 2021/04/29**

**Searched 29.4.21**

1 (Internet or "World wide web" or app or apps or application or smartphone* or phone* or text messag* or SMS or web-based or interactive or video or youtube or whatsapp or facebook or social media or Instagram or Email* or Game* or Gaming or subscription or tweet or twitter or snapchat) in Title or Abstract 9580 results

2 (online NEAR (counsel* or coach* or diar*)) in Title or Abstract 13 results

3 ((remote* or online or digital*) NEAR (deliver* or element* or program* or schedul* or counsel* or advisor* or group* or participa* or tracker*)) in Title or Abstract 372 results

4 e-coach* in Title or Abstract 2 results

5 ((digital* or online* or remote* or tech) NEAR (application* or solution*)) in Title or Abstract 80 results

6 (smart watch* or smartwatch* or wearable* or fittech or software or iphone or i-phone or fitbit or android or mobile device* or vlog* or vlogger* or influencer* or Phone track*) in Title or Abstract 8313 results

7 (Virtual reality NEAR (game* or active* or interface* or system* or simulat* or device* or display* or exercis*)) in Title or Abstract 189 results

8 (VR NEAR (game* or active* or interface* or system* or simulat* or device* or display* or exercis*)) in All text 84 results

9 zoom in All text 3 results

10 (e-diet* or "my fitness pal" or "myfitnesspal") in All text 6 results

11 ((calorie or food) NEAR (diary or diaries or journal* or log* or app* or monitor*)) in All text 522 results

12 ((remote* or online or digital*) NEAR diet*) in All text 20 results

13 #1 or #2 or #3 or #4 or #5 or #6 or #7 or #8 or #9 or #10 or #11 or #12 in All text 17860 results

14 ("Generation Z" or "Gen Z" or "Young person" or "young people" or "Younger generation" or "School age" or "School aged" or school or schools or "School child" or "school children" or "young adult" or "young adults") in All text 12294 results

15 (teen or teens or Teenager* or schoolage* or schoolchild* or freshman or freshmen or sophomore* or Student* or pupil* or adolescen* or Millennial* or "High school" or "Middle school") in All text 10259 results

16 (College* or Universit* or Youth or youths or Boy* or Girl* or "Young man" or "Young men" or "Young woman" or "Young women") in All text 26349 results

17 #14 or #15 or #16 in All text 35753 results

18 ((Unhealthy or non-healthy or fatty or high fat or salty or high sodium or high salt or calorific or high calorie or processed or convenience or healthy or low calorie) NEAR (diet* or meal* or food*)) in All text 594 results

19 ((junk* or ready or TV or television or fast) NEAR/2 (meal* or food*)) in All text 79 results

20 ((poor choice* or good or healthy or balanced or nutritional) NEAR (meal* or food* or meal*)) in All text 345 results

21 #20 or #19 or #18 in All text 826 results

**22 #17 and #21 and #13 in All text 33 results**

**Cochrane Database of Systematic Reviews (CDSR) (Wiley): up to 2021/04/Iss4**

**Searched 28.4.21**

#1 MeSH descriptor: [Internet] explode all trees 4171

#2 MeSH descriptor: [Smartphone] this term only 453

#3 MeSH descriptor: [Wearable Electronic Devices] this term only 87

#4 MeSH descriptor: [Fitness Trackers] this term only 104

#5 MeSH descriptor: [Electronic Mail] this term only 333

#6 MeSH descriptor: [undefined] explode all trees 0

#7 MeSH descriptor: [Video Games] this term only 707

#8 MeSH descriptor: [Virtual Reality Exposure Therapy] this term only 195

#9 MeSH descriptor: [Virtual Reality] this term only 265

#10 MeSH descriptor: [Social Networking] this term only 96

#11 MeSH descriptor: [Online Social Networking] this term only 9

#12 MeSH descriptor: [Mobile Applications] this term only 748

#13 MeSH descriptor: [Telemedicine] this term only 2265

#14 MeSH descriptor: [Self-Help Devices] this term only 137

#15 (Internet or "World wide web" or app or apps or application or smartphone* or phone* or text messag* or SMS or web-based or interactive or video or youtube or whatsapp or facebook or social media or Instagram or Email* or Game* or Gaming or subscription or tweet or twitter or snapchat):ti,ab 111134

#16 (online NEAR/3 (counsel* or coach* or diar*)):ti,ab 363

#17 ((remote* or online or digital*) NEAR/3 (deliver* or element* or program* or schedul* or counsel* or advisor* or group* or participa* or tracker*)):ti,ab 5235

#18 e-coach*:ti,ab 96

#19 ((digital* or online* or remote* or tech) NEAR/3 (application* or solution*)):ti,ab 386

#20 (smart watch* or smartwatch* or wearable* or fittech or software or iphone or i-phone or fitbit or android or mobile device* or vlog* or vlogger* or influencer* or Phone track*):ti,ab 19264

#21 (Virtual reality NEAR/3 (game* or active* or interface* or system* or simulat* or device* or display* or exercis*)):ti,ab 1065

#22 (VR NEAR/3 (game* or active* or interface* or system* or simulat* or device* or display* or exercis*)):ti,ab 599

#23 zoom:ti,ab 235

#24 ((calorie or food) NEAR/3 (diary or diaries or journal* or log* or app* or monitor*)):ti,ab 2993

#25 ((remote* or online or digital*) NEAR/3 diet*):ti,ab 101

#26 (e-diet* or "my fitness pal" or "myfitnesspal"):ti,ab 131

#27 #1 or #2 or #3 or #4 or #5 or #6 or #7 or #8 or #9 or #10 or #11 or #12 or #13 or #14 or #15 or #16 or #17 or #18 or #19 or #20 or #21 or #22 or #23 or #24 or #25 or #26 132304

#28 MeSH descriptor: [Adolescent] explode all trees 105120

#29 MeSH descriptor: [Young Adult] this term only 66544

#30 MeSH descriptor: [Adult] this term only 335465

#31 MeSH descriptor: [Child] explode all trees 56872

#32 MeSH descriptor: [Students] explode all trees 4505

#33 MeSH descriptor: [Schools] this term only 2050

#34 MeSH descriptor: [Universities] this term only 940

#35 ("Generation Z" or "Gen Z" or "Young person" or "young people" or "Younger generation" or "School age" or "School aged" or school or schools or "School child" or "school children" or "young adult" or "young adults"):ti,ab 36521

#36 (teen or teens or Teenager* or schoolage* or schoolchild* or freshman or freshmen or sophomore* or Student* or pupil* or adolescen* or Millennial* or "High school" or "Middle school"):ti,ab 64451

#37 (College* or Universit* or Youth or youths or Boy* or Girl* or "Young man" or "Young men" or "Young woman" or "Young women"):ti,ab 85241

#38 #28 or #29 or #30 or #31 or #32 or #33 or #34 or #35 or #36 or #37 491750

#39 MeSH descriptor: [Fast Foods] this term only 107

#40 ((Unhealthy or non-healthy or fatty or high fat or salty or high sodium or high salt or calorific or high calorie or processed or convenience or healthy or low calorie) NEAR/3 (diet* or meal* or food*)):ti,ab 23064

#41 ((junk* or ready or TV or television or fast) NEAR/2 (meal* or food*)):ti,ab 536

#42 ((poor choice* or good or healthy or balanced or nutritional) NEAR/3 (meal* or food* or meal*)):ti,ab 3188

#43 #39 or #40 or #41 or #42 24389

**#44 #27 and #38 and #43 in Cochrane Reviews, Cochrane Protocols 15**

**Database of Abstracts of Reviews of Effects (DARE) (CRD): up to 2015/03/31**

**Health Technology Assessment Database (HTA) (CRD): up to 2018/03/31**

**Searched 29.3.21**

1 MeSH DESCRIPTOR Internet EXPLODE ALL TREES 257

2 MeSH DESCRIPTOR Smartphone EXPLODE ALL TREES 0

3 MeSH DESCRIPTOR Wearable Electronic Devices 0

4 MeSH DESCRIPTOR Fitness Trackers EXPLODE ALL TREES 0

5 MeSH DESCRIPTOR Electronic Mail EXPLODE ALL TREES 12

6 MeSH DESCRIPTOR Video Games EXPLODE ALL TREES 34

7 MeSH DESCRIPTOR Video Recording EXPLODE ALL TREES 57

8 MeSH DESCRIPTOR Virtual Reality Exposure Therapy EXPLODE ALL TREES 7

9 MeSH DESCRIPTOR Virtual Reality Exposure Therapy EXPLODE ALL TREES 7

10 MeSH DESCRIPTOR Social Media EXPLODE ALL TREES 13

11 MeSH DESCRIPTOR Online Social Networking EXPLODE ALL TREES 0

12 MeSH DESCRIPTOR Mobile Applications EXPLODE ALL TREES 5

13 MeSH DESCRIPTOR Telemedicine EXPLODE ALL TREES 423

14 MeSH DESCRIPTOR Self-Help Devices EXPLODE ALL TREES 72

15 ((Internet or "World wide web" or app or apps or application or smartphone* or phone* or text messag* or SMS or web-based or interactive or video or youtube or whatsapp or facebook or social media or Instagram or Email* or Game* or Gaming or subscription or tweet or twitter or snapchat)) OR ((online NEAR (counsel* or coach* or diar*))) OR (((remote* or online or digital*) NEAR (deliver* or element* or program* or schedul* or counsel* or advisor* or group* or participa* or tracker*))) 4113

16 (e-coach* or zoom) OR (((digital* or online* or remote* or tech) NEAR (application* or solution*))) OR ((smart watch* or smartwatch* or wearable* or fittech or software or iphone or i-phone or fitbit or android or mobile device* or vlog* or vlogger* or influencer* or Phone track*)) 800

17 ((Virtual reality NEAR (game* or active* or interface* or system* or simulat* or device* or display* or exercis*))) OR ((VR NEAR (game* or active* or interface* or system* or simulat* or device* or display* or exercis*)) ) OR ((e-diet* or "my fitness pal" or "myfitnesspal")) 29

18 (((calorie or food) NEAR (diary or diaries or journal* or log* or app* or monitor*)) ) OR (((remote* or online or digital*) NEAR diet*)) 104

19 #1 OR #2 OR #3 OR #4 OR #5 OR #6 OR #7 OR #8 OR #9 OR #10 OR #11 OR #12 OR #13 OR #14 OR #15 OR #16 OR #17 OR #18 5248

20 MeSH DESCRIPTOR Adolescent EXPLODE ALL TREES 4594

21 MeSH DESCRIPTOR Adult 11018

22 MeSH DESCRIPTOR Young Adult 1941

23 MeSH DESCRIPTOR Child EXPLODE ALL TREES 4935

24 MeSH DESCRIPTOR Students EXPLODE ALL TREES 88

25 MeSH DESCRIPTOR Schools EXPLODE ALL TREES 200

26 MeSH DESCRIPTOR Universities EXPLODE ALL TREES 25

27 (("Generation Z" or "Gen Z" or "Young person" or "young people" or "Younger generation" or "School age" or "School aged" or school or schools or "School child" or "school children" or "young adult" or "young adults") ) OR ((teen or teens or Teenager* or schoolage* or schoolchild* or freshman or freshmen or sophomore* or Student* or pupil* or adolescen* or Millennial* or "High school" or "Middle school")) OR ((College* or Universit* or Youth or youths or Boy* or Girl* or "Young man" or "Young men" or "Young woman" or "Young women")) 11581

28 #20 OR #21 OR #22 OR #23 OR #24 OR #25 OR #26 OR #27 19997

29 MeSH DESCRIPTOR Fast Foods EXPLODE ALL TREES 5

30 (((Unhealthy or non-healthy or fatty or high fat or salty or high sodium or high salt or calorific or high calorie or processed or convenience or healthy or low calorie) NEAR (diet* or meal* or food*))) OR (((junk* or ready or TV or television or fast) NEAR (meal* or food*))) OR (((poor choice* or good or healthy or balanced or nutritional) NEAR (meal* or food* or meal*))) 193

31 #29 OR #30 193

**32 (#19 AND #28 AND #31) IN DARE 12**

**33 (#19 AND #28 AND #31) IN HTA 1**

**DARE = 12**

**HTA = 1**

**Epistemonikos (Internet): up to 2021/04/29**

**Searched 29.4.21**

[**https://www.epistemonikos.org/en/advanced_search**](https://www.epistemonikos.org/en/advanced_search)

**Searched Title/Abstract fields:**

1. (Internet or "World wide web" or app or apps or application or smartphone* or phone* or text messag* or SMS or web-based or interactive or video or youtube or whatsapp or facebook or social media or Instagram or Email* or Game* or Gaming or subscription or tweet or twitter or snapchat) OR (online AND (counsel* or coach* or diar*)) OR ((remote* or online or digital*) AND (deliver* or element* or program* or schedul* or counsel* or advisor* or group* or participa* or tracker*)) OR e-coach* OR ((digital* or online* or remote* or tech) AND (application* or solution*)) OR (smart watch* or smartwatch* or wearable* or fittech or software or iphone or i-phone or fitbit or android or mobile device* or vlog* or vlogger* or influencer* or Phone track*) OR (Virtual reality AND (game* or active* or interface* or system* or simulat* or device* or display* or exercis*)) OR (VR AND (game* or active* or interface* or system* or simulat* or device* or display* or exercis*)) OR zoom OR ((calorie or food) AND (diary or diaries or journal* or log* or app* or monitor*)) OR ((remote* or online or digital*) AND diet*) OR (e-diet* or "my fitness pal" or "myfitnesspal")

+

2. ("Generation Z" or "Gen Z" or "Young person" or "young people" or "Younger generation" or "School age" or "School aged" or school or schools or "School child" or "school children" or "young adult" or "young adults") OR (teen or teens or Teenager* or schoolage* or schoolchild* or freshman or freshmen or sophomore* or Student* or pupil* or adolescen* or Millennial* or "High school" or "Middle school") OR (College* or Universit* or Youth or youths or Boy* or Girl* or "Young man" or "Young men" or "Young woman" or "Young women")

+

3. ((Unhealthy or non-healthy or fatty or high fat or salty or high sodium or high salt or calorific or high calorie or processed or convenience or healthy or low calorie) AND (diet* or meal* or food*)) OR ((junk* or ready or TV or television or fast) AND (meal* or food*)) OR ((poor choice* or good or healthy or balanced or nutritional) AND (meal* or food* or meal*))

**Total = 276**

SRs = 61

Broad syntheses = 5

Structured summaries = 1

Primary studies = 209

**Additional focused search for SSBs**

**KSR Evidence (www.ksrevidence.com): up to 2021/08/25**

**Searched 25.8.21**

1 (Internet or "World wide web" or app or apps or application or smartphone* or phone* or text messag* or SMS or web-based or interactive or video or youtube or whatsapp or facebook or social media or Instagram or Email* or Game* or Gaming or subscription or tweet or twitter or snapchat) in Title or Abstract 10454 results

2 (online NEAR (counsel* or coach* or diar*)) in Title or Abstract 14 results

3 ((remote* or online or digital*) NEAR (deliver* or element* or program* or schedul* or counsel* or advisor* or group* or participa* or tracker*)) in Title or Abstract 407 results

4 e-coach* in Title or Abstract 3 results

5 ((digital* or online* or remote* or tech) NEAR (application* or solution*)) in Title or Abstract 92 results

6 (smart watch* or smartwatch* or wearable* or fittech or software or iphone or i-phone or fitbit or android or mobile device* or vlog* or vlogger* or influencer* or Phone track*) in Title or Abstract 8981 results

7 (Virtual reality NEAR (game* or active* or interface* or system* or simulat* or device* or display* or exercis*)) in Title or Abstract 206 results

8 (VR NEAR (game* or active* or interface* or system* or simulat* or device* or display* or exercis*)) in All text 91 results

9 zoom in All text 3 results

10 (e-diet* or "my fitness pal" or "myfitnesspal") in All text 6 results

11 ((calorie or food) NEAR (diary or diaries or journal* or log* or app* or monitor*)) in All text 564 results

12 ((remote* or online or digital*) NEAR diet*) in All text 23 results

13 #1 or #2 or #3 or #4 or #5 or #6 or #7 or #8 or #9 or #10 or #11 or #12 in All text 19381 results

14 ("Generation Z" or "Gen Z" or "Young person" or "young people" or "Younger generation" or "School age" or "School aged" or school or schools or "School child" or "school children" or "young adult" or "young adults") in All text 13723 results

15 (teen or teens or Teenager* or schoolage* or schoolchild* or freshman or freshmen or sophomore* or Student* or pupil* or adolescen* or Millennial* or "High school" or "Middle school") in All text 10934 results

16 (College* or Universit* or Youth or youths or Boy* or Girl* or "Young man" or "Young men" or "Young woman" or "Young women") in All text 30189 results

17 #14 or #15 or #16 in All text 40041 results

18 (sweetened or sugar* or sucrose or fizzy or sport* or energy) NEAR (drink* or beverage* or soda*) in All text 241 results

19 (SSB or SSBs or lemonade* or cola* or softdrink* or soft drink* or fruit juice* or liquid calorie* or chocolate milk or "carbonated drink" or "carbonated drinks") in All text 374 results

20 #18 or #19 in All text 513 results

**21 #13 and #17 and #20 in All text 23 results**

**Cochrane Database of Systematic Reviews (CDSR) (Wiley): up to 2021/08/Iss8**

**Searched 25.8.21**

#1 MeSH descriptor: [Internet] explode all trees 4313

#2 MeSH descriptor: [Smartphone] this term only 530

#3 MeSH descriptor: [Wearable Electronic Devices] this term only 101

#4 MeSH descriptor: [Fitness Trackers] this term only 121

#5 MeSH descriptor: [Electronic Mail] this term only 342

#6 MeSH descriptor: [Video Recording] this term only 1478

#7 MeSH descriptor: [Video Games] this term only 743

#8 MeSH descriptor: [Virtual Reality Exposure Therapy] this term only 202

#9 MeSH descriptor: [Virtual Reality] explode all trees 316

#10 MeSH descriptor: [Social Networking] this term only 109

#11 MeSH descriptor: [Online Social Networking] this term only 9

#12 MeSH descriptor: [Mobile Applications] this term only 864

#13 MeSH descriptor: [Telemedicine] this term only 2414

#14 MeSH descriptor: [Self-Help Devices] this term only 142

#15 (Internet or "World wide web" or app or apps or application or smartphone* or phone* or text messag* or SMS or web-based or interactive or video or youtube or whatsapp or facebook or social media or Instagram or Email* or Game* or Gaming or subscription or tweet or twitter or snapchat):ti,ab 115785

#16 (online NEAR/3 (counsel* or coach* or diar*)):ti,ab 391

#17 ((remote* or online or digital*) NEAR/3 (deliver* or element* or program* or schedul* or counsel* or advisor* or group* or participa* or tracker*)):ti,ab 5626

#18 e-coach*:ti,ab 100

#19 ((digital* or online* or remote* or tech) NEAR/3 (application* or solution*)):ti,ab 435

#20 (smart watch* or smartwatch* or wearable* or fittech or software or iphone or i-phone or fitbit or android or mobile device* or vlog* or vlogger* or influencer* or Phone track*):ti,ab 20375

#21 (Virtual reality NEAR/3 (game* or active* or interface* or system* or simulat* or device* or display* or exercis*)):ti,ab 1121

#22 (VR NEAR/3 (game* or active* or interface* or system* or simulat* or device* or display* or exercis*)):ti,ab 640

#23 zoom:ti,ab 296

#24 ((calorie or food) NEAR/3 (diary or diaries or journal* or log* or app* or monitor*)):ti,ab 3095

#25 ((remote* or online or digital*) NEAR/3 diet*):ti,ab 110

#26 (e-diet* or "my fitness pal" or "myfitnesspal"):ti,ab 142

#27 #1 or #2 or #3 or #4 or #5 or #6 or #7 or #8 or #9 or #10 or #11 or #12 or #13 or #14 or #15 or #16 or #17 or #18 or #19 or #20 or #21 or #22 or #23 or #24 or #25 or #26 138484

#28 MeSH descriptor: [Adolescent] explode all trees 106667

#29 MeSH descriptor: [Young Adult] this term only 68436

#30 MeSH descriptor: [Adult] this term only 340166

#31 MeSH descriptor: [Child] explode all trees 58154

#32 MeSH descriptor: [Students] explode all trees 4706

#33 MeSH descriptor: [Schools] this term only 2166

#34 MeSH descriptor: [Universities] this term only 983

#35 ("Generation Z" or "Gen Z" or "Young person" or "young people" or "Younger generation" or "School age" or "School aged" or school or schools or "School child" or "school children" or "young adult" or "young adults"):ti,ab 37713

#36 (teen or teens or Teenager* or schoolage* or schoolchild* or freshman or freshmen or sophomore* or Student* or pupil* or adolescen* or Millennial* or "High school" or "Middle school"):ti,ab 66581

#37 (College* or Universit* or Youth or youths or Boy* or Girl* or "Young man" or "Young men" or "Young woman" or "Young women"):ti,ab 88196

#38 #28 or #29 or #30 or #31 or #32 or #33 or #34 or #35 or #36 or #37 501555

#39 MeSH descriptor: [Sugar-Sweetened Beverages] explode all trees 30

#40 MeSH descriptor: [Energy Drinks] explode all trees 93

#41 (sweetened or sugar* or sucrose or fizzy or sport* or energy) NEAR/3 (drink* or beverage* or soda*):ti,ab 1979

#42 (SSB or SSBs or lemonade* or cola* or softdrink* or soft drink* or fruit juice* or liquid calorie* or chocolate milk or carbonated drink or carbonated drinks):ti,ab 3365

#43 #39 or #40 or #41 or #42 4897

**#44 #27 and #38 and #43 in Cochrane Reviews, Cochrane Protocols 80**

**CDSR = 80**

**Database of Abstracts of Reviews of Effects (DARE) (CRD): up to 2015/03/31**

**Health Technology Assessment Database (HTA) (CRD): up to 2018/03/31**

**Searched 25.8.21**

1 MeSH DESCRIPTOR Internet EXPLODE ALL TREES 257

2 MeSH DESCRIPTOR Smartphone EXPLODE ALL TREES 0

3 MeSH DESCRIPTOR Wearable Electronic Devices EXPLODE ALL TREES 66

4 MeSH DESCRIPTOR Fitness Trackers EXPLODE ALL TREES 0

5 MeSH DESCRIPTOR Electronic Mail EXPLODE ALL TREES 12

6 MeSH DESCRIPTOR Video Games EXPLODE ALL TREES 34

7 MeSH DESCRIPTOR Video Recording EXPLODE ALL TREES 57

8 MeSH DESCRIPTOR Virtual Reality Exposure Therapy EXPLODE ALL TREES 7

9 MeSH DESCRIPTOR Social Media EXPLODE ALL TREES 13

10 MeSH DESCRIPTOR Online Social Networking EXPLODE ALL TREES 0

11 MeSH DESCRIPTOR Mobile Applications EXPLODE ALL TREES 5

12 MeSH DESCRIPTOR Telemedicine EXPLODE ALL TREES 423

13 MeSH DESCRIPTOR Self-Help Devices EXPLODE ALL TREES 72

14 (((Internet or "World wide web" or app or apps or application or smartphone* or phone* or text messag* or SMS or web-based or interactive or video or youtube or whatsapp or facebook or social media or Instagram or Email* or Game* or Gaming or subscription or tweet or twitter or snapchat)) OR ((online NEAR (counsel* or coach* or diar*))) OR (((remote* or online or digital*) NEAR (deliver* or element* or program* or schedul* or counsel* or advisor* or group* or participa* or tracker*)))) 4113

15 ((e-coach* or zoom) OR (((digital* or online* or remote* or tech) NEAR (application* or solution*))) OR ((smart watch* or smartwatch* or wearable* or fittech or software or iphone or i-phone or fitbit or android or mobile device* or vlog* or vlogger* or influencer* or Phone track*))) 800

16 (((Virtual reality NEAR (game* or active* or interface* or system* or simulat* or device* or display* or exercis*))) OR ((VR NEAR (game* or active* or interface* or system* or simulat* or device* or display* or exercis*)) ) OR ((e-diet* or "my fitness pal" or "myfitnesspal"))) 29

17 ((((calorie or food) NEAR (diary or diaries or journal* or log* or app* or monitor*)) ) OR (((remote* or online or digital*) NEAR diet*))) 104

18 (#1 OR #2 OR #3 OR #4 OR #5 OR #6 OR #7 OR #8 OR #9 OR #10 OR #11 OR #12 OR #13 OR #14 OR #15 OR #16 OR #17) 5309

19 MeSH DESCRIPTOR Adolescent EXPLODE ALL TREES 4594

20 MeSH DESCRIPTOR Adult 11018

21 MeSH DESCRIPTOR Young Adult 1941

22 MeSH DESCRIPTOR Child EXPLODE ALL TREES 4935

23 MeSH DESCRIPTOR Students EXPLODE ALL TREES 88

24 MeSH DESCRIPTOR Schools EXPLODE ALL TREES 200

25 MeSH DESCRIPTOR Universities EXPLODE ALL TREES 25

26 ((("Generation Z" or "Gen Z" or "Young person" or "young people" or "Younger generation" or "School age" or "School aged" or school or schools or "School child" or "school children" or "young adult" or "young adults") ) OR ((teen or teens or Teenager* or schoolage* or schoolchild* or freshman or freshmen or sophomore* or Student* or pupil* or adolescen* or Millennial* or "High school" or "Middle school")) OR ((College* or Universit* or Youth or youths or Boy* or Girl* or "Young man" or "Young men" or "Young woman" or "Young women"))) 11581

27 #19 OR #20 OR #21 OR #22 OR #23 OR #24 OR #25 OR #26 19997

28 MeSH DESCRIPTOR Sugar-Sweetened Beverages EXPLODE ALL TREES 0

29 MeSH DESCRIPTOR Energy Drinks EXPLODE ALL TREES 0

30 ((sweetened or sugar* or sucrose or fizzy or sport* or energy) NEAR (drink* or beverage* or soda*)) 12

31 ((SSB or SSBs or lemonade* or cola* or softdrink* or soft drink* or fruit juice* or liquid calorie* or chocolate milk or carbonated drink or carbonated drinks)) 115

32 #28 OR #29 OR #30 OR #31 127

33 #18 AND #27 AND #32 5

**34 (#33) IN DARE 2**

**35 (#33) IN HTA 3**

**Epistemonikos (Internet): up to 2021/08/26**

**Searched 26.8.21**

[**https://www.epistemonikos.org/en/advanced_search**](https://www.epistemonikos.org/en/advanced_search)

**Searched Title/Abstract fields:**

1. (Internet or "World wide web" or app or apps or application or smartphone* or phone* or text messag* or SMS or web-based or interactive or video or youtube or whatsapp or facebook or social media or Instagram or Email* or Game* or Gaming or subscription or tweet or twitter or snapchat) OR (online AND (counsel* or coach* or diar*)) OR ((remote* or online or digital*) AND (deliver* or element* or program* or schedul* or counsel* or advisor* or group* or participa* or tracker*)) OR e-coach* OR ((digital* or online* or remote* or tech) AND (application* or solution*)) OR (smart watch* or smartwatch* or wearable* or fittech or software or iphone or i-phone or fitbit or android or mobile device* or vlog* or vlogger* or influencer* or Phone track*) OR (Virtual reality AND (game* or active* or interface* or system* or simulat* or device* or display* or exercis*)) OR (VR AND (game* or active* or interface* or system* or simulat* or device* or display* or exercis*)) OR zoom OR ((calorie or food) AND (diary or diaries or journal* or log* or app* or monitor*)) OR ((remote* or online or digital*) AND diet*) OR (e-diet* or "my fitness pal" or "myfitnesspal")

+

2. ("Generation Z" or "Gen Z" or "Young person" or "young people" or "Younger generation" or school or schools or "young adult" or "young adults") OR (teen or teens or Teenager* or schoolage* or schoolchild* or freshman or freshmen or sophomore* or Student* or pupil* or adolescen* or Millennial*) OR (College* or Universit* or Youth or youths or Boy* or Girl* or "Young man" or "Young men" or "Young woman" or "Young women")

+

3. ((sweetened or sugar* or sucrose or fizzy or sport* or energy) and (drink* or beverage* or soda*)) or SSB or SSBs or lemonade* or cola* or softdrink* or "soft drink*" or "fruit juice*" or "liquid calorie*" or "chocolate milk" or "carbonated drink" or "carbonated drinks"

**Total = 259**

SRs = 48

Broad syntheses =2

Structured summaries = 0

Primary studies = 210

**Digital interventions for physical inactivity**

| **Database** | **Dates** | **Results** |
| --- | --- | --- |
| KSR Evidence | up to 2021/04/29 | 359 |
| CDSR | up to 2021/04/Iss4 | 230 |
| DARE | up to 2015/03/31 | 12 |
| HTA | up to 2018/03/31 | 25 |
| Epistemonikos | up to 2021/04/30 | 1925 |
| **Total** | | **2551** |

**KSR Evidence (www.ksrevidence.com): up to 2021/04/29**

**Searched 29.4.21**

1 (Internet or "World wide web" or app or apps or application or smartphone* or phone* or text messag* or SMS or web-based or interactive or video or youtube or whatsapp or facebook or social media or Instagram or Email* or Game* or Gaming or subscription or twitter or tweet* or snapchat) in Title or Abstract 9702 results

2 (online NEAR (counsel* or coach* or diar*)) in Title or Abstract 13 results

3 ((remote* or online or digital*) NEAR (deliver* or element* or program* or schedul* or Counsel* or advisor* or group* or participa* or tracker*)) in Title or Abstract 372 results

4 e-coach* or zoom in Title or Abstract 5 results

5 ((digital* or online* or remote* or tech) NEAR (application* or solution*)) in Title or Abstract 80 results

6 (smart watch* or smartwatch* or wearable* or fittech or software or iphone or mobile device* or vlog* or vlogger* or influencer* or Phone track* or i-phone or android) in Title or Abstract 8313 results

7 (Virtual reality NEAR (game* or active* or interface* or system* or simulat* or device* or display* or exercis*)) in Title or Abstract 189 results

8 (VR NEAR (game* or active* or interface* or system* or simulat* or device* or display* or exercis*)) in Title or Abstract 82 results

9 ((step or steps or fitness or activ* or exercis* or digital*) NEAR (count* or device* or monitor* or app or apps or tracker*)) in Title or Abstract 779 results

10 (Daily NEAR (step or steps or walk*)) in Title or Abstract 79 results

11 (((remote* or online or digital* or "technology assisted") NEAR fitness) or tracker*) in Title or Abstract 60 results

12 (activometer or "active-o-meter" or personal tracker*) in Title or Abstract 4 results

13 (Peloton or "e-fitness" or "e-activit*" or fitbit or garmin or "pokemon go" or "apple health" or strava or "7 minute workout" or "30 day fitness" or "Wii-fit" or pedometer* or runkeeper or mapmyfitness or myfitnesspal or acceleromet* or IMU or "inertial measurement unit") in Title or Abstract 284 results

14 ((Activity or Fitness) NEAR challeng*) in Title or Abstract 28 results

15 #1 or #2 or #3 or #4 or #5 or #6 or #7 or #8 or #9 or #10 or #11 or #12 or #13 or #14 in All text 18256 results

16 ("Generation Z" or "Gen Z" or "Young person" or "young people" or "Younger generation" or "School age" or "School child" or "school children" or "young adult" or " young adults" or "School aged" or school or schools) in Title or Abstract 3721 results

17 (teen or teens or Teenager* or schoolage* or schoolchild* or freshman or freshmen or sophomore* or Student* or pupil* or adolescen* or Millennial* or "High school" or "Middle school") in Title or Abstract 7135 results

18 (College* or Universit* or Youth or youths or Boy* or Girl* or "Young man" or "Young men" or "Young woman" or "Young women") in Title or Abstract 7920 results

19 #16 or #17 or #18 in All text 15145 results

20 (Exercis* or walk* or cycl* or bike or bikes or run or running or jog or jogging or dance* or dancing or aerobics or swim or swimming) in Title or Abstract 10103 results

21 (physical* NEAR (inactiv* or activ*)) in Title or Abstract 3707 results

22 (Playground* or play-ground* or playarea* or play-area* or Active* play* or gym or gyms) in Title or Abstract 248 results

23 ("walking bus" or "walk to school" or "walk to work" or "daily mile" or "park and stride") in Title or Abstract 1 result

24 Sedentary in Title or Abstract 618 results

25 (Step NEAR (count* or goal* or target*)) in Title or Abstract 89 results

26 activity level* in Title or Abstract 2251 results

27 ((time or pattern*) NEAR (activ* or inactiv*)) in Title or Abstract 736 results

28 (Hourly prompt* or activity remind* or physical education) in Title or Abstract 790 results

29 #20 or #21 or #22 or #23 or #24 or #25 or #26 or #27 or #28 in All text 14570 results

**30 #19 and #15 and #29 in All text 359 results**

**Cochrane Database of Systematic Reviews (CDSR) (Wiley): up to 2021/04/Iss4**

**Searched 29.4.21**

#1 MeSH descriptor: [Internet] explode all trees 4171

#2 MeSH descriptor: [Smartphone] this term only 453

#3 MeSH descriptor: [Wearable Electronic Devices] this term only 87

#4 MeSH descriptor: [Fitness Trackers] this term only 104

#5 MeSH descriptor: [Electronic Mail] this term only 333

#6 MeSH descriptor: [Video Recording] this term only 1441

#7 MeSH descriptor: [Video Games] this term only 707

#8 MeSH descriptor: [Virtual Reality Exposure Therapy] this term only 195

#9 MeSH descriptor: [Online Social Networking] this term only 9

#10 MeSH descriptor: [Social Networking] this term only 96

#11 MeSH descriptor: [Computers, Handheld] explode all trees 730

#12 MeSH descriptor: [Mobile Applications] this term only 748

#13 MeSH descriptor: [Telemedicine] this term only and with qualifier(s): [instrumentation - IS, methods - MT] 1288

#14 MeSH descriptor: [Self-Help Devices] this term only 137

#15 MeSH descriptor: [Remote Sensing Technology] this term only 45

#16 MeSH descriptor: [Monitoring, Ambulatory] this term only 554

#17 MeSH descriptor: [Actigraphy] this term only 489

#18 MeSH descriptor: [Wireless Technology] this term only 43

#19 (Internet or "World wide web" or app or apps or application or smartphone* or phone* or text messag* or SMS or web-based or interactive or video or youtube or whatsapp or facebook or social media or Instagram or Email* or Game* or Gaming or subscription or twitter or tweet* or snapchat):ti,ab 111339

#20 (online NEAR/3 (counsel* or coach* or diar*)):ti,ab 363

#21 ((remote* or online or digital*) NEAR/3 (deliver* or element* or program* or schedul* or Counsel* or advisor* or group* or participa* or tracker*)):ti,ab 5235

#22 e-coach*:ti,ab 96

#23 ((digital* or online* or remote* or tech) NEAR/3 (application* or solution*)):ti,ab 386

#24 (smart watch* or smartwatch* or wearable* or fittech or software or iphone or mobile device* or vlog* or vlogger* or influencer* or Phone track* or i-phone or android):ti,ab 18936

#25 (Virtual reality NEAR/3 (game* or active* or interface* or system* or simulat* or device* or display* or exercis*)):ti,ab 1065

#26 (VR NEAR/3 (game* or active* or interface* or system* or simulat* or device* or display* or exercis*)):ti,ab 599

#27 zoom:ti,ab 235

#28 ((step or steps or fitness or activ* or exercis* or digital*) NEAR/3 (count* or device* or monitor* or app or apps or tracker*)):ti,ab 8040

#29 (Daily NEAR/2 (step or steps or walk*)):ti,ab 1212

#30 (((remote* or online or digital* or "technology assisted") NEAR/3 fitness) or tracker*):ti,ab 906

#31 (activometer or "active-o-meter" or personal tracker*):ti,ab 72

#32 (Peloton or "e-fitness" or "e-activit*" or fitbit or garmin or "pokemon go" or "apple health" or strava or "7 minute workout" or "30 day fitness" or "Wii-fit" or pedometer* or runkeeper or mapmyfitness or myfitnesspal or acceleromet* or IMU or "inertial measurement unit"):ti,ab 6193

#33 ((Activity or Fitness) NEAR/3 challeng*):ti,ab 136

#34 #1 or #2 or #3 or #4 or #5 or #6 or #7 or #8 or #9 or #10 or #11 or #12 or #13 or #14 or #15 or #16 or #17 or #18 or #19 or #20 or #21 or #22 or #23 or #24 or #25 or #26 or #27 or #28 or #29 or #30 or #31 or #32 or #33 139792

#35 MeSH descriptor: [Adolescent] explode all trees 105120

#36 MeSH descriptor: [Adult] this term only 335465

#37 MeSH descriptor: [Young Adult] this term only 66544

#38 MeSH descriptor: [Child] explode all trees 56872

#39 MeSH descriptor: [Students] explode all trees 4505

#40 MeSH descriptor: [Schools] explode all trees 3128

#41 MeSH descriptor: [Universities] explode all trees 940

#42 ("Generation Z" or "Gen Z" or "Young person" or "young people" or "Younger generation" or "School age" or "School child" or "school children" or "young adult" or " young adults" or "School aged" or school or schools):ti,ab 36521

#43 (teen or teens or Teenager* or schoolage* or schoolchild* or freshman or freshmen or sophomore* or Student* or pupil* or adolescen* or Millennial* or "High school" or "Middle school"):ti,ab 64451

#44 (College* or Universit* or Youth or youths or Boy* or Girl* or "Young man" or "Young men" or "Young woman" or "Young women"):ti,ab 85241

#45 #35 or #36 or #37 or #38 or #39 or #40 or #41 or #42 or #43 or #44 491754

#46 MeSH descriptor: [Physical Fitness] this term only 2928

#47 MeSH descriptor: [Cardiorespiratory Fitness] this term only 294

#48 MeSH descriptor: [Walking] this term only 4191

#49 MeSH descriptor: [Walking Speed] this term only 167

#50 MeSH descriptor: [Stair Climbing] this term only 15

#51 MeSH descriptor: [Exercise] explode all trees 25218

#52 MeSH descriptor: [Sports] explode all trees 15873

#53 MeSH descriptor: [Sedentary Behavior] this term only 1155

#54 (Exercis* or walk* or cycl* or bike or bikes or run or running or jog or jogging or dance* or dancing or aerobics or swim or swimming):ti,ab 194886

#55 (physical* NEAR/2 (inactiv* or activ*)):ti,ab 31929

#56 (Playground* or play-ground* or playarea* or play-area* or Active* play* or gym or gyms):ti,ab 3759

#57 ("walking bus" or "walk to school" or "walk to work" or "daily mile" or "park and stride"):ti,ab 14

#58 Sedentary:ti,ab 7349

#59 (Step NEAR/3 (count* or goal* or target*)):ti,ab 1240

#60 activity level*:ti,ab 48003

#61 ((time or pattern*) NEAR/2 (activ* or inactiv*)):ti,ab 4469

#62 (Hourly prompt* or activity remind* or physical education):ti,ab 9866

#63 #46 or #47 or #48 or #49 or #50 or #51 or #52 or #53 or #54 or #55 or #56 or #57 or #58 or #59 or #60 or #61 or #62 252890

**#64 #34 and #45 and #63 in Cochrane Reviews, Cochrane Protocols 230**

**Database of Abstracts of Reviews of Effects (DARE) (CRD): up to 2015/03/31**

**Health Technology Assessment Database (HTA) (CRD): up to 2018/03/31**

**Searched 29.4.21**

1 MeSH DESCRIPTOR Internet EXPLODE ALL TREES 257

2 MeSH DESCRIPTOR Smartphone EXPLODE ALL TREES 0

3 MeSH DESCRIPTOR Wearable Electronic Devices 0

4 MeSH DESCRIPTOR Fitness Trackers 0

5 MeSH DESCRIPTOR Electronic Mail 12

6 MeSH DESCRIPTOR Video Recording 34

7 MeSH DESCRIPTOR Video Games 34

8 MeSH DESCRIPTOR Virtual Reality Exposure Therapy 7

9 MeSH DESCRIPTOR Online Social Networking EXPLODE ALL TREES 0

10 MeSH DESCRIPTOR Social Networking EXPLODE ALL TREES 6

11 MeSH DESCRIPTOR Computers, Handheld EXPLODE ALL TREES 13

12 MeSH DESCRIPTOR Mobile Applications 5

13 MeSH DESCRIPTOR Telemedicine EXPLODE ALL TREES WITH QUALIFIER MT 103

14 MeSH DESCRIPTOR Telemedicine EXPLODE ALL TREES WITH QUALIFIER IS 15

15 MeSH DESCRIPTOR Self-Help Devices 39

16 MeSH DESCRIPTOR Remote Sensing Technology 8

17 MeSH DESCRIPTOR Monitoring, Ambulatory 66

18 MeSH DESCRIPTOR Actigraphy 9

19 MeSH DESCRIPTOR Wireless Technology 7

20 ((Internet or "World wide web" or app or apps or application or smartphone* or phone* or text messag* or SMS or web-based or interactive or video or youtube or whatsapp or facebook or social media or Instagram or Email* or Game* or Gaming or subscription or twitter or tweet* or snapchat)) OR ((online NEAR (counsel* or coach* or diar*))) OR (((remote* or online or digital*) NEAR (deliver* or element* or program* or schedul* or Counsel* or advisor* or group* or participa* or tracker*))) IN HTA 1255

21 (e-coach* or zoom) OR (((digital* or online* or remote* or tech) NEAR (application* or solution*))) OR ((smart watch* or smartwatch* or wearable* or fittech or software or iphone or mobile device* or vlog* or vlogger* or influencer* or Phone track* or i-phone or android)) IN HTA 84

22 ((Virtual reality NEAR (game* or active* or interface* or system* or simulat* or device* or display* or exercis*))) OR ((VR NEAR (game* or active* or interface* or system* or simulat* or device* or display* or exercis*))) OR (((step or steps or fitness or activ* or exercis* or digital*) NEAR (count* or device* or monitor* or app or apps or tracker*))) IN HTA 48

23 ((Daily NEAR (step or steps or walk*))) OR ((((remote* or online or digital* or "technology assisted") NEAR fitness) or tracker*)) OR ((activometer or "active-o-meter" or personal tracker*)) IN HTA 5

24 ((activometer or "active-o-meter" or personal tracker*)) OR ((Peloton or "e-fitness" or "e-activit*" or fitbit or garmin or "pokemon go" or "apple health" or strava or "7 minute workout" or "30 day fitness" or "Wii-fit" or pedometer* or runkeeper or mapmyfitness or myfitnesspal or acceleromet* or IMU or "inertial measurement unit")) OR (((Activity or Fitness) NEAR challeng*)) IN HTA 9

25 #1 OR #2 OR #3 OR #4 OR #5 OR #6 OR #7 OR #8 OR #9 OR #10 OR #11 OR #12 OR #13 OR #14 OR #15 OR #16 OR #17 OR #18 OR #19 OR #20 OR #21 OR #22 OR #23 OR #24 1857

26 MeSH DESCRIPTOR Adolescent EXPLODE ALL TREES 4594

27 MeSH DESCRIPTOR Adult 11018

28 MeSH DESCRIPTOR Young Adult 1941

29 MeSH DESCRIPTOR Child EXPLODE ALL TREES 4935

30 MeSH DESCRIPTOR Students EXPLODE ALL TREES 88

31 MeSH DESCRIPTOR Schools EXPLODE ALL TREES 200

32 MeSH DESCRIPTOR Universities EXPLODE ALL TREES 25

33 (("Generation Z" or "Gen Z" or "Young person" or "young people" or "Younger generation" or "School age" or "School child" or "school children" or "young adult" or " young adults" or "School aged" or school or schools)) OR ((teen or teens or Teenager* or schoolage* or schoolchild* or freshman or freshmen or sophomore* or Student* or pupil* or adolescen* or Millennial* or "High school" or "Middle school")) OR ((College* or Universit* or Youth or youths or Boy* or Girl* or "Young man" or "Young men" or "Young woman" or "Young women")) IN HTA 1434

34 #26 OR #27 OR #28 OR #29 OR #30 OR #31 OR #32 OR #33 16171

35 MeSH DESCRIPTOR Physical Fitness 166

36 MeSH DESCRIPTOR Cardiorespiratory Fitness 0

37 MeSH DESCRIPTOR Walking 211

38 MeSH DESCRIPTOR Walking Speed 1

39 MeSH DESCRIPTOR Stair Climbing 0

40 MeSH DESCRIPTOR Exercise EXPLODE ALL TREES 1137

41 MeSH DESCRIPTOR Sports EXPLODE ALL TREES 552

42 MeSH DESCRIPTOR Sedentary Behavior 0

43 ((Exercis* or walk* or cycl* or bike or bikes or run or running or jog or jogging or dance* or dancing or aerobics or swim or swimming)) OR ((physical* NEAR (inactiv* or activ*))) OR ((Playground* or play-ground* or playarea* or play-area* or Active* play* or gym or gyms)) IN HTA 907

44 (activity level*) OR (((time or pattern*) NEAR (activ* or inactiv*))) OR ((Hourly prompt* or activity remind* or physical education)) IN HTA 44

45 #35 OR #36 OR #37 OR #38 OR #39 OR #40 OR #41 OR #42 OR #43 OR #44 2232

46 #25 AND #34 AND #45 42

**47 (#25 AND #34 AND #45) IN HTA 25**

**48 (#25 AND #34 AND #45) IN DARE 12**

**DARE = 12**

**HTA = 25**

**Epistemonikos (Internet): up to 2021/04/30**

**Searched 30.4.21**

[**https://www.epistemonikos.org/en/advanced_search**](https://www.epistemonikos.org/en/advanced_search)

**Search 1**

**Title only:**

(Internet or "World wide web" or app or apps or application or smartphone* or phone* or text messag* or SMS or web-based or interactive or video or youtube or whatsapp or facebook or social media or Instagram or Email* or Game* or Gaming or subscription or twitter or tweet* or snapchat) OR (online AND (counsel* or coach* or diar*)) OR ((remote* or online or digital*) AND (deliver* or element* or program* or schedul* or Counsel* or advisor* or group* or participa* or tracker*)) OR e-coach* or zoom OR ((digital* or online* or remote* or tech) AND (application* or solution*)) OR (smart watch* or smartwatch* or wearable* or fittech or software or iphone or mobile device* or vlog* or vlogger* or influencer* or Phone track* or i-phone or android) OR (Virtual reality AND (game* or active* or interface* or system* or simulat* or device* or display* or exercis*)) OR (VR AND (game* or active* or interface* or system* or simulat* or device* or display* or exercis*)) OR ((step or steps or fitness or activ* or exercis* or digital*) AND (count* or device* or monitor* or app or apps or tracker*)) OR (Daily AND (step or steps or walk*)) OR (((remote* or online or digital* or "technology assisted") AND fitness) or tracker*) OR (activometer or "active-o-meter" or personal tracker*) OR (Peloton or "e-fitness" or "e-activit*" or fitbit or garmin or "pokemon go" or "apple health" or strava or "7 minute workout" or "30 day fitness" or "Wii-fit" or pedometer* or runkeeper or mapmyfitness or myfitnesspal or acceleromet* or IMU or "inertial measurement unit") OR ((Activity or Fitness) AND challeng*)

+

**Title/Abstract:**

("Generation Z" or "Gen Z" or "Young person" or "young people" or "Younger generation" or "School age" or "School child" or "school children" or "young adult" or " young adults" or "School aged" or school or schools) OR (teen or teens or Teenager* or schoolage* or schoolchild* or freshman or freshmen or sophomore* or Student* or pupil* or adolescen* or Millennial* or "High school" or "Middle school") OR (College* or Universit* or Youth or youths or Boy* or Girl* or "Young man" or "Young men" or "Young woman" or "Young women")

+

**Title/Abstract:**

(Exercis* or walk* or cycl* or bike or bikes or run or running or jog or jogging or dance* or dancing or aerobics or swim or swimming) OR (physical* AND (inactiv* or activ*))

**Total = 666**

SRs = 151

Broad syntheses = 6

Structured summaries = 5

Primary studies = 504

**Search 2**

**Title only:**

(Internet or "World wide web" or app or apps or application or smartphone* or phone* or text messag* or SMS or web-based or interactive or video or youtube or whatsapp or facebook or social media or Instagram or Email* or Game* or Gaming or subscription or twitter or tweet* or snapchat) OR (online AND (counsel* or coach* or diar*)) OR ((remote* or online or digital*) AND (deliver* or element* or program* or schedul* or Counsel* or advisor* or group* or participa* or tracker*)) OR e-coach* or zoom OR ((digital* or online* or remote* or tech) AND (application* or solution*)) OR (smart watch* or smartwatch* or wearable* or fittech or software or iphone or mobile device* or vlog* or vlogger* or influencer* or Phone track* or i-phone or android) OR (Virtual reality AND (game* or active* or interface* or system* or simulat* or device* or display* or exercis*)) OR (VR AND (game* or active* or interface* or system* or simulat* or device* or display* or exercis*)) OR ((step or steps or fitness or activ* or exercis* or digital*) AND (count* or device* or monitor* or app or apps or tracker*)) OR (Daily AND (step or steps or walk*)) OR (((remote* or online or digital* or "technology assisted") AND fitness) or tracker*) OR (activometer or "active-o-meter" or personal tracker*) OR (Peloton or "e-fitness" or "e-activit*" or fitbit or garmin or "pokemon go" or "apple health" or strava or "7 minute workout" or "30 day fitness" or "Wii-fit" or pedometer* or runkeeper or mapmyfitness or myfitnesspal or acceleromet* or IMU or "inertial measurement unit") OR ((Activity or Fitness) AND challeng*)

+

**Title/Abstract:**

("Generation Z" or "Gen Z" or "Young person" or "young people" or "Younger generation" or "School age" or "School child" or "school children" or "young adult" or " young adults" or "School aged" or school or schools) OR (teen or teens or Teenager* or schoolage* or schoolchild* or freshman or freshmen or sophomore* or Student* or pupil* or adolescen* or Millennial* or "High school" or "Middle school") OR (College* or Universit* or Youth or youths or Boy* or Girl* or "Young man" or "Young men" or "Young woman" or "Young women")

+

**Title/Abstract:**

(Playground* or play-ground* or playarea* or play-area* or Active* play* or gym or gyms) OR ("walking bus" or "walk to school" or "walk to work" or "daily mile" or "park and stride") OR Sedentary OR (Step AND (count* or goal* or target*)) OR activity level*

**Total = 936**

SRs = 193

Broad syntheses = 5

Structured summaries = 1

Primary studies = 737

**Search 3**

**Title only:**

(Internet or "World wide web" or app or apps or application or smartphone* or phone* or text messag* or SMS or web-based or interactive or video or youtube or whatsapp or facebook or social media or Instagram or Email* or Game* or Gaming or subscription or twitter or tweet* or snapchat) OR (online AND (counsel* or coach* or diar*)) OR ((remote* or online or digital*) AND (deliver* or element* or program* or schedul* or Counsel* or advisor* or group* or participa* or tracker*)) OR e-coach* or zoom OR ((digital* or online* or remote* or tech) AND (application* or solution*)) OR (smart watch* or smartwatch* or wearable* or fittech or software or iphone or mobile device* or vlog* or vlogger* or influencer* or Phone track* or i-phone or android) OR (Virtual reality AND (game* or active* or interface* or system* or simulat* or device* or display* or exercis*)) OR (VR AND (game* or active* or interface* or system* or simulat* or device* or display* or exercis*)) OR ((step or steps or fitness or activ* or exercis* or digital*) AND (count* or device* or monitor* or app or apps or tracker*)) OR (Daily AND (step or steps or walk*)) OR (((remote* or online or digital* or "technology assisted") AND fitness) or tracker*) OR (activometer or "active-o-meter" or personal tracker*) OR (Peloton or "e-fitness" or "e-activit*" or fitbit or garmin or "pokemon go" or "apple health" or strava or "7 minute workout" or "30 day fitness" or "Wii-fit" or pedometer* or runkeeper or mapmyfitness or myfitnesspal or acceleromet* or IMU or "inertial measurement unit") OR ((Activity or Fitness) AND challeng*)

+

**Title/Abstract:**

("Generation Z" or "Gen Z" or "Young person" or "young people" or "Younger generation" or "School age" or "School child" or "school children" or "young adult" or " young adults" or "School aged" or school or schools) OR (teen or teens or Teenager* or schoolage* or schoolchild* or freshman or freshmen or sophomore* or Student* or pupil* or adolescen* or Millennial* or "High school" or "Middle school") OR (College* or Universit* or Youth or youths or Boy* or Girl* or "Young man" or "Young men" or "Young woman" or "Young women")

+

**Title/Abstract:**

((time or pattern*) AND (activ* or inactiv*)) OR (Hourly prompt* or activity remind* or physical education)

**Total = 323**

**SRs = 65**

**Broad syntheses = 1**

**Structured summaries = 0**

**Primary studies = 257**

**Overall Total = 1925**

Overall SRs = 409

Overall Broad syntheses = 12

Overall Structured summaries = 6

Overall Primary studies = 1498

**Stage2: Focussed Rapid review searches**

**Digital interventions for** **alcohol consumption**

| **Database** | **Dates** | **Results** |
| --- | --- | --- |
| Embase | 2016-2021/06/22 | 2026 |
| MEDLINE & In-Process | 2016-2021/06/22 | 1347 |
| MEDLINE DU & AoP | up to 2021/06/22 | 83 |
| **Total** | | **3456** |

**Embase (Ovid): 2016-2021/06/22**

**Searched 22.6.21**

Digital Interventions + Young People + alcohol consumption + Prevent/Promote (No A, 2016-C)

1 exp Internet/ (116669)

2 exp smart watch/ (3946)

3 exp Mobile application/ (16132)

4 Self care software/ (13)

5 social media/ (27555)

6 e-mail/ (24339)

7 exp mobile phone/ (34425)

8 exp video game/ (4307)

9 wearable computer/ (665)

10 video game console/ (121)

11 online social networking/ (526)

12 Electronic device/ (6028)

13 monitor/ or personal monitor/ (7915)

14 exp mobile application/ (16132)

15 Remote sensing/ (10667)

16 telemedicine/ or teletherapy/ (32303)

17 Assistive technology/ (2080)

18 (Internet or "World wide web" or app or apps or application or smartphone$ or phone$ or text messag$ or SMS or web-based or interactive or video or youtube or whatsapp or facebook or social media or Instagram or Email$ or Game$ or Gaming or subscription or twitter or tweet$ or snapchat).ti,ab,ot. (1553729)

19 (online adj3 (counsel$ or coach$ or diar$)).ti,ab,ot. (796)

20 ((remote$ or online or digital$) adj3 (deliver$ or element$ or program$ or schedul$ or advisor$ or group$ or participa$ or tracker$)).ti,ab,ot. (21522)

21 e-coach$.ti,ab,ot. (68)

22 ((digital$ or online$ or remote$ or tech) adj3 (application$ or solution$)).ti,ab,ot. (5737)

23 (smart watch$ or smartwatch$ or wearable$ or fittech or software or iphone or mobile device$ or vlog$ or vlogger$ or influencer$ or Phone track$ or i-phone or android).ti,ab,ot. (341079)

24 (Virtual reality adj3 (game$ or active$ or interface$ or system$ or simulat$ or device$ or display$ or exercis$)).ti,ab,ot. (4679)

25 (VR adj3 (game$ or active$ or interface$ or system$ or simulat$ or device$ or display$ or exercis$)).ti,ab,ot. (2554)

26 zoom.ti,ab,ot. (3010)

27 or/1-26 (1969669)

28 exp adolescent/ (1595771)

29 young adult/ (411036)

30 child/ or boy/ or girl/ or school child/ (1980201)

31 school/ or college/ or community college/ or high school/ or medical school/ or middle school/ or pharmacy school/ or primary school/ or university/ (363010)

32 ("Generation Z" or "Gen Z" or "Young person" or "young people" or "Younger generation" or "School age" or "School child" or "school children" or "young adult" or "young adults" or "School aged" or school or schools).ti,ab,ot. (503873)

33 (teen or teens or Teenager$ or schoolage$ or schoolchild$ or freshman or freshmen or sophomore$ or Student$ or pupil$ or adolescen$ or Millennial$ or "High school" or "Middle school").ti,ab,ot. (841386)

34 (College$ or Universit$ or Youth or youths or Boy$ or Girl$ or "Young man" or "Young men" or "Young woman" or "Young women").ti,ab,ot. (1368337)

35 or/28-34 (4481529)

36 alcohol consumption/ (137244)

37 exp alcoholic beverage/ (31308)

38 binge drinking/ (6118)

39 drinking behaviour/ (50444)

40 (alcohol or beer or beers or spirits or liquor or wine or "binge drinking" or "problem drinker" or "binge drinker" or "problem drinking").ti,ab,ot. (405074)

41 ((problem$ or excessive$ or excess) adj3 (alcohol or drink$)).ti,ab,ot. (27872)

42 or/36-41 (461210)

43 animal/ or animal experiment/ (4188878)

44 (rat or rats or mouse or mice or murine or rodent or rodents or hamster or hamsters or pig or pigs or porcine or rabbit or rabbits or animal or animals or dogs or dog or cats or cow or bovine or sheep or ovine or monkey or monkeys).ti,ab,ot,hw. (7017023)

45 or/43-44 (7017023)

46 exp human/ or human experiment/ (22449055)

47 45 not (45 and 46) (5344219)

48 27 and 35 and 42 (7362)

49 48 not 47 (7224)

50 limit 49 to yr="2016 -Current" (3278)

51 exp *Prevention/ or prevent$.ti,ab,ot. (2320004)

52 (Risk adj3 (reduc$ or lower$ or adjust$ or decrease$)).ti,ab,ot. (384898)

53 exp *health education/ (116193)

54 *health behavior/ (25779)

55 *Motivation/ (29051)

56 (promot$ or program$ or campaign$ or strateg$ or educat$ or literacy or attitude$ or polic$ or encourage$ or awareness or Mentoring or advertis$).ti,ab,ot. (4913856)

57 (Health adj3 (behaviour$ or behavior$)).ti,ab,ot. (65089)

58 or/51-57 (6857213)

59 50 and 58 (2031)

60 (letter or editorial or note).pt. (2733035)

**61 59 not 60 (2026)**

**Medline & In-Process Citations (Ovid): 2016-2021/06/22**

**Searched 22.6.21**

1 Accelerometry/ (6195)

2 Fitness Trackers/ (775)

3 Wearable Electronic Devices/ (4127)

4 Wireless Technology/ (3861)

5 Telemedicine/is, mt [Instrumentation, Methods] (10466)

6 Mobile Applications/ (7918)

7 exp Internet/ (85251)

8 smartphone/ (6023)

9 Wearable electronic devices/ (4127)

10 Fitness trackers/ (775)

11 Electronic mail/ (2778)

12 Video recording/ (26184)

13 Video game/ (6023)

14 virtual reality therapy/ (701)

15 virtual reality system/ (0)

16 (Internet or "World wide web" or app or apps or application or smartphone$ or phone$ or text messag$ or SMS or web-based or interactive or video or youtube or whatsapp or facebook or social media or Instagram or Email$ or Game$ or Gaming or subscription or twitter or tweet$ or snapchat).ti,ab,ot. (1204948)

17 (online adj3 (counsel$ or coach$ or diar$)).ti,ab,ot. (517)

18 ((remote$ or online or digital$) adj3 (deliver$ or element$ or program$ or schedul$ or advisor$ or group$ or participa$ or tracker$)).ti,ab,ot. (14558)

19 e-coach$.ti,ab,ot. (55)

20 ((digital$ or online$ or remote$ or tech) adj3 (application$ or solution$)).ti,ab,ot. (4616)

21 (smart watch$ or smartwatch$ or wearable$ or fittech or software or iphone or mobile device$ or vlog$ or vlogger$ or influencer$ or Phone track$ or i-phone or android).ti,ab,ot. (202686)

22 (Virtual reality adj3 (game$ or active$ or interface$ or system$ or simulat$ or device$ or display$ or exercis$)).ti,ab,ot. (3348)

23 (VR adj3 (game$ or active$ or interface$ or system$ or simulat$ or device$ or display$ or exercis$)).ti,ab,ot. (1754)

24 zoom.ti,ab,ot. (1899)

25 or/1-24 (1450022)

26 exp adolescent/ (2096278)

27 young adult/ (922721)

28 exp child/ or exp student/ (2098806)

29 schools/ or universities/ (84566)

30 ("Generation Z" or "Gen Z" or "Young person" or "young people" or "Younger generation" or "School age" or "School child" or "school children" or "young adult" or " young adults" or "School aged" or school or schools).ti,ab,ot. (390525)

31 (teen or teens or Teenager$ or schoolage$ or schoolchild$ or freshman or freshmen or sophomore$ or Student$ or pupil$ or adolescen$ or Millennial$ or "High school" or "Middle school").ti,ab,ot. (623820)

32 (College$ or Universit$ or Youth or youths or Boy$ or Girl$ or "Young man" or "Young men" or "Young woman" or "Young women").ti,ab,ot. (838743)

33 or/26-32 (4356798)

34 exp alcoholic beverages/ (21259)

35 exp alcohol drinking/ (71773)

36 (alcohol or beer or beers or spirits or liquor or wine or "binge drinking" or "problem drinker" or "binge drinker" or "problem drinking").ti,ab,ot. (295345)

37 ((problem$ or excessive$ or excess) adj3 (alcohol or drink$)).ti,ab,ot. (19724)

38 or/34-37 (316172)

39 exp animals/ not (exp animals/ and humans/) (4839846)

40 25 and 33 and 38 (4910)

41 40 not 39 (4890)

42 limit 41 to yr="2016 -Current" (2114)

43 Prevent$.ti,ab,ot. (1481238)

44 (Risk adj3 (reduc$ or lower$ or adjust$ or decrease$)).ti,ab,ot. (257414)

45 exp *Health Education/ (148494)

46 exp *Health Behavior/ (176720)

47 exp *School Health Services/ or *motivation/ or *drive/ or *goals/ (52987)

48 (promot$ or program$ or campaign$ or strateg$ or educat$ or literacy or attitude$ or polic$ or encourage$ or awareness or Mentoring or advertis$).ti,ab,ot. (3753088)

49 ((Health$ or chang$) adj3 (behaviour$ or behavior$)).ti,ab,ot. (119979)

50 or/43-49 (5161189)

**51 42 and 50 (1347)**

**Medline Daily Update and ePubs Ahead of Print (Ovid): 2016-2021/06/22**

**Searched 22.6.21**

1 Accelerometry/ (40)

2 Fitness Trackers/ (13)

3 Wearable Electronic Devices/ (56)

4 Wireless Technology/ (8)

5 Telemedicine/is, mt [Instrumentation, Methods] (49)

6 Mobile Applications/ (94)

7 exp Internet/ (274)

8 smartphone/ (94)

9 Wearable electronic devices/ (56)

10 Fitness trackers/ (13)

11 Electronic mail/ (1)

12 Video recording/ (58)

13 Video game/ (31)

14 virtual reality therapy/ (2)

15 virtual reality system/ (0)

16 (Internet or "World wide web" or app or apps or application or smartphone$ or phone$ or text messag$ or SMS or web-based or interactive or video or youtube or whatsapp or facebook or social media or Instagram or Email$ or Game$ or Gaming or subscription or twitter or tweet$ or snapchat).ti,ab,ot. (29728)

17 (online adj3 (counsel$ or coach$ or diar$)).ti,ab,ot. (36)

18 ((remote$ or online or digital$) adj3 (deliver$ or element$ or program$ or schedul$ or advisor$ or group$ or participa$ or tracker$)).ti,ab,ot. (940)

19 e-coach$.ti,ab,ot. (1)

20 ((digital$ or online$ or remote$ or tech) adj3 (application$ or solution$)).ti,ab,ot. (182)

21 (smart watch$ or smartwatch$ or wearable$ or fittech or software or iphone or mobile device$ or vlog$ or vlogger$ or influencer$ or Phone track$ or i-phone or android).ti,ab,ot. (5983)

22 (Virtual reality adj3 (game$ or active$ or interface$ or system$ or simulat$ or device$ or display$ or exercis$)).ti,ab,ot. (126)

23 (VR adj3 (game$ or active$ or interface$ or system$ or simulat$ or device$ or display$ or exercis$)).ti,ab,ot. (91)

24 zoom.ti,ab,ot. (97)

25 or/1-24 (35632)

26 exp adolescent/ (3885)

27 young adult/ (3415)

28 exp child/ or exp student/ (6340)

29 schools/ or universities/ (361)

30 ("Generation Z" or "Gen Z" or "Young person" or "young people" or "Younger generation" or "School age" or "School child" or "school children" or "young adult" or " young adults" or "School aged" or school or schools).ti,ab,ot. (10111)

31 (teen or teens or Teenager$ or schoolage$ or schoolchild$ or freshman or freshmen or sophomore$ or Student$ or pupil$ or adolescen$ or Millennial$ or "High school" or "Middle school").ti,ab,ot. (18489)

32 (College$ or Universit$ or Youth or youths or Boy$ or Girl$ or "Young man" or "Young men" or "Young woman" or "Young women").ti,ab,ot. (19976)

33 or/26-32 (43083)

34 exp alcoholic beverages/ (48)

35 exp alcohol drinking/ (130)

36 (alcohol or beer or beers or spirits or liquor or wine or "binge drinking" or "problem drinker" or "binge drinker" or "problem drinking").ti,ab,ot. (5667)

37 ((problem$ or excessive$ or excess) adj3 (alcohol or drink$)).ti,ab,ot. (430)

38 or/34-37 (5700)

39 exp animals/ not (exp animals/ and humans/) (9668)

40 25 and 33 and 38 (138)

41 40 not 39 (138)

42 limit 41 to yr="2016 -Current" (122)

43 Prevent$.ti,ab,ot. (31185)

44 (Risk adj3 (reduc$ or lower$ or adjust$ or decrease$)).ti,ab,ot. (8025)

45 exp *Health Education/ (176)

46 exp *Health Behavior/ (390)

47 exp *School Health Services/ or *motivation/ or *drive/ or *goals/ (105)

48 (promot$ or program$ or campaign$ or strateg$ or educat$ or literacy or attitude$ or polic$ or encourage$ or awareness or Mentoring or advertis$).ti,ab,ot. (95960)

49 ((Health$ or chang$) adj3 (behaviour$ or behavior$)).ti,ab,ot. (3815)

50 or/43-49 (121521)

**51 42 and 50 (83)**

**Digital interventions for Unhealthy Diet**

| **Database** | **Dates** | **Results for unhealthy diet** | **Results for SSBs** |
| --- | --- | --- | --- |
| Embase | 2015-2021/06/22 | 1394 | 524 |
| MEDLINE & In-Process | 2015-2021/06/22 | 745 | 325 |
| MEDLINE DU & AoP | up to 2021/06/22 | 49 | 14 |
| **Total** | | **2188** | **864** |

**Embase (Ovid): 2015-2021/06/22**

**Searched: 23.6.21**

Digital Interventions + Young People + Unhealthy Diet (No A, 2015-C)

1 exp *Internet/ (38550)

2 exp *smart watch/ (1084)

3 exp *Mobile application/ (8245)

4 *Self care software/ (5)

5 exp *social media/ (10179)

6 *e-mail/ (1637)

7 exp *mobile phone/ (13839)

8 exp *video game/ (1853)

9 *wearable computer/ (285)

10 *video game console/ (31)

11 *personal monitor/ (226)

12 *Electronic device/ (2670)

13 *online social networking/ (215)

14 *monitor/ (1354)

15 exp *mobile application/ (8245)

16 *telemedicine/ or *teletherapy/ (16875)

17 *Assistive technology/ (1004)

18 *Remote sensing/ (4008)

19 (Internet or "World wide web" or app or apps or application or smartphone$ or phone$ or text messag$ or SMS or web-based or interactive or video or youtube or whatsapp or facebook or social media or Instagram or Email$ or Game$ or Gaming or subscription or tweet or twitter or snapchat).ti,ab,ot. (1552459)

20 (online adj3 (counsel$ or coach$ or diar$)).ti,ab,ot. (796)

21 ((remote$ or online or digital$) adj3 (deliver$ or element$ or program$ or schedul$ or counsel$ or advisor$ or group$ or participa$ or tracker$)).ti,ab,ot. (21882)

22 e-coach$.ti,ab,ot. (68)

23 ((digital$ or online$ or remote$ or tech) adj3 (application$ or solution$)).ti,ab,ot. (5737)

24 (smart watch$ or smartwatch$ or wearable$ or fittech or software or iphone or i-phone or fitbit or android or mobile device$ or vlog$ or vlogger$ or influencer$ or Phone track$).ti,ab,ot. (341844)

25 (Virtual reality adj3 (game$ or active$ or interface$ or system$ or simulat$ or device$ or display$ or exercis$)).ti,ab,ot. (4679)

26 (VR adj3 (game$ or active$ or interface$ or system$ or simulat$ or device$ or display$ or exercis$)).ti,ab,ot. (2554)

27 zoom.ti,ab,ot. (3010)

28 ((calorie or food) adj3 (diary or diaries or journal$ or log$ or app$ or monitor$)).ti,ab,ot. (28176)

29 ((remote$ or online or digital$) adj3 diet$).ti,ab,ot. (390)

30 (e-diet$ or "my fitness pal" or runkeeper or mapmyfitness or myfitnesspal or acceleromet$ or IMU or "inertial measurement unit").ti,ab,ot. (25149)

31 or/1-30 (1936757)

32 exp *adolescent/ (27919)

33 *young adult/ (2849)

34 *child/ or *boy/ or *girl/ or *school child/ (94247)

35 school/ or college/ or community college/ or high school/ or medical school/ or middle school/ or pharmacy school/ or primary school/ or university/ (363010)

36 ("Generation Z" or "Gen Z" or "Young person" or "young people" or "Younger generation" or "School age" or "School aged" or school or schools or "School child" or "school children" or "young adult" or "young adults").ti,ab,ot. (503873)

37 (teen or teens or Teenager$ or schoolage$ or schoolchild$ or freshman or freshmen or sophomore$ or Student$ or pupil$ or adolescen$ or Millennial$ or "High school" or "Middle school").ti,ab,ot. (841386)

38 (College$ or Universit$ or Youth or youths or Boy$ or Girl$ or "Young man" or "Young men" or "Young woman" or "Young women").ti,ab,ot. (1368337)

39 or/32-38 (2377483)

40 *unhealthy diet/ (204)

41 *high calorie diet/ (180)

42 *high salt diet/ (218)

43 *low carbohydrate diet/ (1322)

44 *caloric intake/ (10563)

45 *caloric density/ (51)

46 *meal skipping/ (29)

47 *junk food/ (95)

48 *fast food/ (1954)

49 ((Unhealthy or non-healthy or fatty or high fat or salty or high sodium or high salt or calorific or high calorie or processed or convenience or healthy or low calorie) adj3 (diet$ or meal$ or food$)).ti,ab,ot,hw. (114287)

50 ((junk$ or ready or TV or television or fast) adj2 (meal$ or food$)).ti,ab,ot,hw. (12378)

51 ((poor choice$ or good or healthy or balanced or nutritional) adj3 (meal$ or food$ or meal$)).ti,ab,ot,hw. (16328)

52 or/40-51 (139702)

53 animal/ or animal experiment/ (4188878)

54 (rat or rats or mouse or mice or murine or rodent or rodents or hamster or hamsters or pig or pigs or porcine or rabbit or rabbits or animal or animals or dogs or dog or cats or cow or bovine or sheep or ovine or monkey or monkeys).ti,ab,ot,hw. (7017023)

55 or/53-54 (7017023)

56 exp human/ or human experiment/ (22449055)

57 55 not (55 and 56) (5344219)

58 31 and 39 and 52 (2348)

59 58 not 57 (2308)

**60 limit 59 to yr="2015 -Current" (1394)**

**Medline and In-Process, In-Data-Review & Other Non-Indexed Citations: 2015-2021/06/22**

**Searched: 23.6.21**

1 exp *Internet/ (46947)

2 *smartphone/ (3965)

3 *Wearable electronic devices/ (3120)

4 *Fitness trackers/ (473)

5 *Electronic mail/ (1388)

6 *Video recording/ (7782)

7 *Video game/ (4578)

8 *virtual reality therapy/ (626)

9 *virtual reality system/ (0)

10 *Social Media/ (8118)

11 *social networking/ or *online social networking/ (2862)

12 *Mobile Applications/ (6183)

13 *Telemedicine/ (23617)

14 *Self-Help Devices/ (3735)

15 (Internet or "World wide web" or app or apps or application or smartphone$ or phone$ or text messag$ or SMS or web-based or interactive or video or youtube or whatsapp or facebook or social media or Instagram or Email$ or Game$ or Gaming or subscription or tweet or twitter or snapchat).ti,ab,ot. (1203763)

16 (online adj3 (counsel$ or coach$ or diar$)).ti,ab,ot. (517)

17 ((remote$ or online or digital$) adj3 (deliver$ or element$ or program$ or schedul$ or counsel$ or advisor$ or group$ or participa$ or tracker$)).ti,ab,ot. (14799)

18 e-coach$.ti,ab,ot. (55)

19 ((digital$ or online$ or remote$ or tech) adj3 (application$ or solution$)).ti,ab,ot. (4616)

20 (smart watch$ or smartwatch$ or wearable$ or fittech or software or iphone or i-phone or fitbit or android or mobile device$ or vlog$ or vlogger$ or influencer$ or Phone track$).ti,ab,ot. (203144)

21 (Virtual reality adj3 (game$ or active$ or interface$ or system$ or simulat$ or device$ or display$ or exercis$)).ti,ab,ot. (3348)

22 (VR adj3 (game$ or active$ or interface$ or system$ or simulat$ or device$ or display$ or exercis$)).ti,ab,ot. (1754)

23 zoom.ti,ab,ot. (1899)

24 ((calorie or food) adj3 (diary or diaries or journal$ or log$ or app$ or monitor$)).ti,ab,ot. (22341)

25 ((remote$ or online or digital$) adj3 diet$).ti,ab,ot. (258)

26 (e-diet$ or "my fitness pal" or "myfitnesspal").ti,ab,ot. (463)

27 or/1-26 (1443071)

28 exp *adolescent/ (5548)

29 *young adult/ (102)

30 exp *child/ or exp *student/ (94446)

31 schools/ or universities/ (84566)

32 ("Generation Z" or "Gen Z" or "Young person" or "young people" or "Younger generation" or "School age" or "School aged" or school or schools or "School child" or "school children" or "young adult" or "young adults").ti,ab,ot. (390525)

33 (teen or teens or Teenager$ or schoolage$ or schoolchild$ or freshman or freshmen or sophomore$ or Student$ or pupil$ or adolescen$ or Millennial$ or "High school" or "Middle school").ti,ab,ot. (623820)

34 (College$ or Universit$ or Youth or youths or Boy$ or Girl$ or "Young man" or "Young men" or "Young woman" or "Young women").ti,ab,ot. (838743)

35 or/28-34 (1506715)

36 *Fast Foods/ (1431)

37 ((Unhealthy or non-healthy or fatty or high fat or salty or high sodium or high salt or calorific or high calorie or processed or convenience or healthy or low calorie) adj3 (diet$ or meal$ or food$)).ti,ab,ot,hw. (82229)

38 ((junk$ or ready or TV or television or fast) adj2 (meal$ or food$)).ti,ab,ot,hw. (6328)

39 ((poor choice$ or good or healthy or balanced or nutritional) adj3 (meal$ or food$ or meal$)).ti,ab,ot,hw. (11968)

40 or/36-39 (91064)

41 exp animals/ not (exp animals/ and humans/) (4839846)

42 27 and 35 and 40 (1133)

43 42 not 41 (1122)

**44 limit 43 to yr="2015 -Current" (745)**

**Medline Daily Update and Epubs Ahead-of-Print (Ovid): up to 2021/06/22**

**Searched: 23.6.21**

1 exp *Internet/ (151)

2 *smartphone/ (42)

3 *Wearable electronic devices/ (51)

4 *Fitness trackers/ (6)

5 *Electronic mail/ (1)

6 *Video recording/ (19)

7 *Video game/ (26)

8 *virtual reality therapy/ (2)

9 *virtual reality system/ (0)

10 *Social Media/ (95)

11 *social networking/ or *online social networking/ (12)

12 *Mobile Applications/ (82)

13 *Telemedicine/ (198)

14 *Self-Help Devices/ (5)

15 (Internet or "World wide web" or app or apps or application or smartphone$ or phone$ or text messag$ or SMS or web-based or interactive or video or youtube or whatsapp or facebook or social media or Instagram or Email$ or Game$ or Gaming or subscription or tweet or twitter or snapchat).ti,ab,ot. (29647)

16 (online adj3 (counsel$ or coach$ or diar$)).ti,ab,ot. (36)

17 ((remote$ or online or digital$) adj3 (deliver$ or element$ or program$ or schedul$ or counsel$ or advisor$ or group$ or participa$ or tracker$)).ti,ab,ot. (962)

18 e-coach$.ti,ab,ot. (1)

19 ((digital$ or online$ or remote$ or tech) adj3 (application$ or solution$)).ti,ab,ot. (182)

20 (smart watch$ or smartwatch$ or wearable$ or fittech or software or iphone or i-phone or fitbit or android or mobile device$ or vlog$ or vlogger$ or influencer$ or Phone track$).ti,ab,ot. (6008)

21 (Virtual reality adj3 (game$ or active$ or interface$ or system$ or simulat$ or device$ or display$ or exercis$)).ti,ab,ot. (126)

22 (VR adj3 (game$ or active$ or interface$ or system$ or simulat$ or device$ or display$ or exercis$)).ti,ab,ot. (91)

23 zoom.ti,ab,ot. (97)

24 ((calorie or food) adj3 (diary or diaries or journal$ or log$ or app$ or monitor$)).ti,ab,ot. (650)

25 ((remote$ or online or digital$) adj3 diet$).ti,ab,ot. (9)

26 (e-diet$ or "my fitness pal" or "myfitnesspal").ti,ab,ot. (4)

27 or/1-26 (36078)

28 exp *adolescent/ (1)

29 *young adult/ (0)

30 exp *child/ or exp *student/ (632)

31 schools/ or universities/ (361)

32 ("Generation Z" or "Gen Z" or "Young person" or "young people" or "Younger generation" or "School age" or "School aged" or school or schools or "School child" or "school children" or "young adult" or "young adults").ti,ab,ot. (10111)

33 (teen or teens or Teenager$ or schoolage$ or schoolchild$ or freshman or freshmen or sophomore$ or Student$ or pupil$ or adolescen$ or Millennial$ or "High school" or "Middle school").ti,ab,ot. (18489)

34 (College$ or Universit$ or Youth or youths or Boy$ or Girl$ or "Young man" or "Young men" or "Young woman" or "Young women").ti,ab,ot. (19976)

35 or/28-34 (36671)

36 *Fast Foods/ (6)

37 ((Unhealthy or non-healthy or fatty or high fat or salty or high sodium or high salt or calorific or high calorie or processed or convenience or healthy or low calorie) adj3 (diet$ or meal$ or food$)).ti,ab,ot,hw. (1962)

38 ((junk$ or ready or TV or television or fast) adj2 (meal$ or food$)).ti,ab,ot,hw. (134)

39 ((poor choice$ or good or healthy or balanced or nutritional) adj3 (meal$ or food$ or meal$)).ti,ab,ot,hw. (376)

40 or/36-39 (2173)

41 exp animals/ not (exp animals/ and humans/) (9668)

42 27 and 35 and 40 (52)

43 42 not 41 (52)

**44 limit 43 to yr="2015 -Current" (49)**

**Additional focussed search for SSBs**

**Embase (Ovid): 2015-2021/08/24**

**Searched: 25.8.21**

Digital Interventions + Young People + SSBs (No A, 2015-C)

1 exp *Internet/ (38738)

2 exp *smart watch/ (1109)

3 exp *Mobile application/ (8560)

4 *Self care software/ (5)

5 exp *social media/ (10727)

6 *e-mail/ (1638)

7 exp *mobile phone/ (14125)

8 exp *video game/ (1912)

9 *wearable computer/ (309)

10 *video game console/ (33)

11 *personal monitor/ (221)

12 *Electronic device/ (2799)

13 *online social networking/ (215)

14 *monitor/ (1355)

15 exp *mobile application/ (8560)

16 *telemedicine/ or *teletherapy/ (17450)

17 *Assistive technology/ (997)

18 *Remote sensing/ (4104)

19 (Internet or "World wide web" or app or apps or application or smartphone$ or phone$ or text messag$ or SMS or web-based or interactive or video or youtube or whatsapp or facebook or social media or Instagram or Email$ or Game$ or Gaming or subscription or tweet or twitter or snapchat).ti,ab,ot. (1570659)

20 (online adj3 (counsel$ or coach$ or diar$)).ti,ab,ot. (813)

21 ((remote$ or online or digital$) adj3 (deliver$ or element$ or program$ or schedul$ or counsel$ or advisor$ or group$ or participa$ or tracker$)).ti,ab,ot. (22560)

22 e-coach$.ti,ab,ot. (68)

23 ((digital$ or online$ or remote$ or tech) adj3 (application$ or solution$)).ti,ab,ot. (5882)

24 (smart watch$ or smartwatch$ or wearable$ or fittech or software or iphone or i-phone or fitbit or android or mobile device$ or vlog$ or vlogger$ or influencer$ or Phone track$).ti,ab,ot. (347414)

25 (Virtual reality adj3 (game$ or active$ or interface$ or system$ or simulat$ or device$ or display$ or exercis$)).ti,ab,ot. (4742)

26 (VR adj3 (game$ or active$ or interface$ or system$ or simulat$ or device$ or display$ or exercis$)).ti,ab,ot. (2620)

27 zoom.ti,ab,ot. (3149)

28 ((calorie or food) adj3 (diary or diaries or journal$ or log$ or app$ or monitor$)).ti,ab,ot. (28569)

29 ((remote$ or online or digital$) adj3 diet$).ti,ab,ot. (399)

30 (e-diet$ or "my fitness pal" or runkeeper or mapmyfitness or myfitnesspal or acceleromet$ or IMU or "inertial measurement unit").ti,ab,ot. (25491)

31 or/1-30 (1961337)

32 exp *adolescent/ (27939)

33 *young adult/ (2849)

34 *child/ or *boy/ or *girl/ or *school child/ (94234)

35 school/ or college/ or community college/ or high school/ or medical school/ or middle school/ or pharmacy school/ or primary school/ or university/ (365028)

36 ("Generation Z" or "Gen Z" or "Young person" or "young people" or "Younger generation" or "School age" or "School aged" or school or schools or "School child" or "school children" or "young adult" or "young adults").ti,ab,ot. (508685)

37 (teen or teens or Teenager$ or schoolage$ or schoolchild$ or freshman or freshmen or sophomore$ or Student$ or pupil$ or adolescen$ or Millennial$ or "High school" or "Middle school").ti,ab,ot. (850004)

38 (College$ or Universit$ or Youth or youths or Boy$ or Girl$ or "Young man" or "Young men" or "Young woman" or "Young women").ti,ab,ot. (1380538)

39 or/32-38 (2397856)

40 exp sweetened beverage/ or sports drink/ or energy drink/ (5404)

41 ((sweetened or sugar$ or sucrose or fizzy or sport$ or energy) adj3 (drink$ or beverage$ or soda$)).ti,ab,ot. (12188)

42 (SSB or SSBs or lemonade$ or cola$ or softdrink$ or soft drink$ or fruit juice$ or liquid calorie$ or chocolate milk or "carbonated drink" or "carbonated drinks").ti,ab. (24465)

43 or/40-42 (34596)

44 animal/ or animal experiment/ (4214717)

45 (rat or rats or mouse or mice or murine or rodent or rodents or hamster or hamsters or pig or pigs or porcine or rabbit or rabbits or animal or animals or dogs or dog or cats or cow or bovine or sheep or ovine or monkey or monkeys).ti,ab,ot,hw. (7046816)

46 or/44-45 (7046816)

47 exp human/ or human experiment/ (22627583)

48 46 not (46 and 47) (5359875)

49 31 and 39 and 43 (966)

50 49 not 48 (956)

**51 limit 50 to yr="2015 -Current" (524)**

**Medline and In-Process, In-Data-Review & Other Non-Indexed Citations: 2015-2021/08/24**

**Searched 25.8.21**

1 exp *Internet/ (47920)

2 *smartphone/ (4147)

3 *Wearable electronic devices/ (3421)

4 *Fitness trackers/ (505)

5 *Electronic mail/ (1410)

6 *Video recording/ (7866)

7 *Video game/ (4730)

8 *virtual reality therapy/ (642)

9 *virtual reality system/ (0)

10 *Social Media/ (8762)

11 *social networking/ or *online social networking/ (2948)

12 *Mobile Applications/ (6571)

13 *Telemedicine/ (24644)

14 *Self-Help Devices/ (3790)

15 (Internet or "World wide web" or app or apps or application or smartphone$ or phone$ or text messag$ or SMS or web-based or interactive or video or youtube or whatsapp or facebook or social media or Instagram or Email$ or Game$ or Gaming or subscription or tweet or twitter or snapchat).ti,ab,ot. (1225131)

16 (online adj3 (counsel$ or coach$ or diar$)).ti,ab,ot. (535)

17 ((remote$ or online or digital$) adj3 (deliver$ or element$ or program$ or schedul$ or counsel$ or advisor$ or group$ or participa$ or tracker$)).ti,ab,ot. (15366)

18 e-coach$.ti,ab,ot. (55)

19 ((digital$ or online$ or remote$ or tech) adj3 (application$ or solution$)).ti,ab,ot. (4759)

20 (smart watch$ or smartwatch$ or wearable$ or fittech or software or iphone or i-phone or fitbit or android or mobile device$ or vlog$ or vlogger$ or influencer$ or Phone track$).ti,ab,ot. (207859)

21 (Virtual reality adj3 (game$ or active$ or interface$ or system$ or simulat$ or device$ or display$ or exercis$)).ti,ab,ot. (3425)

22 (VR adj3 (game$ or active$ or interface$ or system$ or simulat$ or device$ or display$ or exercis$)).ti,ab,ot. (1813)

23 zoom.ti,ab,ot. (1975)

24 ((calorie or food) adj3 (diary or diaries or journal$ or log$ or app$ or monitor$)).ti,ab,ot. (22846)

25 ((remote$ or online or digital$) adj3 diet$).ti,ab,ot. (267)

26 (e-diet$ or "my fitness pal" or "myfitnesspal").ti,ab,ot. (465)

27 or/1-26 (1470091)

28 exp *adolescent/ (5550)

29 *young adult/ (103)

30 exp *child/ or exp *student/ (96868)

31 schools/ or universities/ (86885)

32 ("Generation Z" or "Gen Z" or "Young person" or "young people" or "Younger generation" or "School age" or "School aged" or school or schools or "School child" or "school children" or "young adult" or "young adults").ti,ab,ot. (395823)

33 (teen or teens or Teenager$ or schoolage$ or schoolchild$ or freshman or freshmen or sophomore$ or Student$ or pupil$ or adolescen$ or Millennial$ or "High school" or "Middle school").ti,ab,ot. (633079)

34 (College$ or Universit$ or Youth or youths or Boy$ or Girl$ or "Young man" or "Young men" or "Young woman" or "Young women").ti,ab,ot. (849850)

35 or/28-34 (1526532)

36 exp Sugar-Sweetened Beverages/ (466)

37 exp Energy Drinks/ (832)

38 ((sweetened or sugar$ or sucrose or fizzy or sport$ or energy) adj3 (drink$ or beverage$ or soda$)).ti,ab,ot. (8952)

39 (SSB or SSBs or lemonade$ or cola$ or softdrink$ or soft drink$ or fruit juice$ or liquid calorie$ or chocolate milk or "carbonated drink" or "carbonated drinks").ti,ab. (18998)

40 or/36-39 (25682)

41 27 and 35 and 40 (568)

42 exp animals/ not (exp animals/ and humans/) (4873671)

43 41 not 42 (566)

**44 limit 43 to yr="2015 -Current" (325)**

**Medline Epub Ahead of Print (Ovid): 2015-2021/08/24**

**Medline Daily Update (Ovid): 2015-2021/08/24**

**Searched: 25.8.21**

1 exp *Internet/ (126)

2 *smartphone/ (24)

3 *Wearable electronic devices/ (56)

4 *Fitness trackers/ (8)

5 *Electronic mail/ (4)

6 *Video recording/ (15)

7 *Video game/ (16)

8 *virtual reality therapy/ (0)

9 *virtual reality system/ (0)

10 *Social Media/ (89)

11 *social networking/ or *online social networking/ (15)

12 *Mobile Applications/ (72)

13 *Telemedicine/ (173)

14 *Self-Help Devices/ (11)

15 (Internet or "World wide web" or app or apps or application or smartphone$ or phone$ or text messag$ or SMS or web-based or interactive or video or youtube or whatsapp or facebook or social media or Instagram or Email$ or Game$ or Gaming or subscription or tweet or twitter or snapchat).ti,ab,ot. (28507)

16 (online adj3 (counsel$ or coach$ or diar$)).ti,ab,ot. (38)

17 ((remote$ or online or digital$) adj3 (deliver$ or element$ or program$ or schedul$ or counsel$ or advisor$ or group$ or participa$ or tracker$)).ti,ab,ot. (1033)

18 e-coach$.ti,ab,ot. (1)

19 ((digital$ or online$ or remote$ or tech) adj3 (application$ or solution$)).ti,ab,ot. (204)

20 (smart watch$ or smartwatch$ or wearable$ or fittech or software or iphone or i-phone or fitbit or android or mobile device$ or vlog$ or vlogger$ or influencer$ or Phone track$).ti,ab,ot. (5582)

21 (Virtual reality adj3 (game$ or active$ or interface$ or system$ or simulat$ or device$ or display$ or exercis$)).ti,ab,ot. (120)

22 (VR adj3 (game$ or active$ or interface$ or system$ or simulat$ or device$ or display$ or exercis$)).ti,ab,ot. (96)

23 zoom.ti,ab,ot. (123)

24 ((calorie or food) adj3 (diary or diaries or journal$ or log$ or app$ or monitor$)).ti,ab,ot. (635)

25 ((remote$ or online or digital$) adj3 diet$).ti,ab,ot. (9)

26 (e-diet$ or "my fitness pal" or "myfitnesspal").ti,ab,ot. (4)

27 or/1-26 (34626)

28 exp *adolescent/ (0)

29 *young adult/ (0)

30 exp *child/ or exp *student/ (320)

31 schools/ or universities/ (333)

32 ("Generation Z" or "Gen Z" or "Young person" or "young people" or "Younger generation" or "School age" or "School aged" or school or schools or "School child" or "school children" or "young adult" or "young adults").ti,ab,ot. (10132)

33 (teen or teens or Teenager$ or schoolage$ or schoolchild$ or freshman or freshmen or sophomore$ or Student$ or pupil$ or adolescen$ or Millennial$ or "High school" or "Middle school").ti,ab,ot. (18411)

34 (College$ or Universit$ or Youth or youths or Boy$ or Girl$ or "Young man" or "Young men" or "Young woman" or "Young women").ti,ab,ot. (19561)

35 or/28-34 (36103)

36 exp Sugar-Sweetened Beverages/ (9)

37 exp Energy Drinks/ (3)

38 ((sweetened or sugar$ or sucrose or fizzy or sport$ or energy) adj3 (drink$ or beverage$ or soda$)).ti,ab,ot. (267)

39 (SSB or SSBs or lemonade$ or cola$ or softdrink$ or soft drink$ or fruit juice$ or liquid calorie$ or chocolate milk or "carbonated drink" or "carbonated drinks").ti,ab. (425)

40 or/36-39 (603)

41 27 and 35 and 40 (18)

42 exp animals/ not (exp animals/ and humans/) (4369)

43 41 not 42 (18)

**44 limit 43 to yr="2015 -Current" (15)**

**Digital interventions for physical inactivity**

| **Database** | **Dates** | **Results** |
| --- | --- | --- |
| Embase | 2015-2021/06/21 | 7965 |
| MEDLINE & In-Process | 2015-2021/06/21 | 4976 |
| MEDLINE DU & AoP | up to 2021/06/21 | 281 |
| **Total** | | **13222** |

**Embase (Ovid): 2015-2021/06/21**

**Searched 22.6.21**

Digital Interventions + Young People + Exercise/Sedentary + Prevent/Promote (No A, 2015-C)

1 exp *Internet/ (38548)

2 exp *smart watch/ (1084)

3 exp *Mobile application/ (8243)

4 *Self care software/ (5)

5 *social media/ (10171)

6 *e-mail/ (1637)

7 exp *mobile phone/ (13834)

8 exp *video game/ (1852)

9 *wearable computer/ (285)

10 *video game console/ (31)

11 *online social networking/ (215)

12 *Tablet computer/ (891)

13 *Electronic device/ (2670)

14 *monitor/ (1354)

15 exp *activity tracker/ (994)

16 *telemedicine/ or *teletherapy/ (16869)

17 *Assistive technology/ (1004)

18 *Remote sensing/ (4006)

19 *personal monitor/ (226)

20 (Internet or "World wide web" or app or apps or application or smartphone$ or phone$ or text messag$ or SMS or web-based or interactive or video or youtube or whatsapp or facebook or social media or Instagram or Email$ or Game$ or Gaming or subscription or twitter or tweet$ or snapchat).ti,ab,ot. (1553413)

21 (online adj3 (counsel$ or coach$ or diar$)).ti,ab,ot. (796)

22 ((remote$ or online or digital$) adj3 (deliver$ or element$ or program$ or schedul$ or advisor$ or group$ or participa$ or tracker$)).ti,ab,ot. (21514)

23 e-coach$.ti,ab,ot. (68)

24 ((digital$ or online$ or remote$ or tech) adj3 (application$ or solution$)).ti,ab,ot. (5733)

25 (smart watch$ or smartwatch$ or wearable$ or fittech or software or iphone or mobile device$ or vlog$ or vlogger$ or influencer$ or Phone track$ or i-phone or android).ti,ab,ot. (340997)

26 (Virtual reality adj3 (game$ or active$ or interface$ or system$ or simulat$ or device$ or display$ or exercis$)).ti,ab,ot. (4675)

27 (VR adj3 (game$ or active$ or interface$ or system$ or simulat$ or device$ or display$ or exercis$)).ti,ab,ot. (2553)

28 zoom.ti,ab,ot. (3008)

29 (Daily adj2 (step or steps or walk$)).ti,ab,ot. (3621)

30 ((step or steps or fitness or activ$ or exercis$ or digital$) adj3 (count$ or device$ or monitor$ or app or apps or tracker$)).ti,ab,ot. (68259)

31 (((remote$ or online or digital$ or "technology assisted") adj3 fitness) or tracker$).ti,ab,ot. (7363)

32 (activometer or "active-o-meter" or personal tracker$).ti,ab,ot. (1)

33 (Peloton or "e-fitness" or "e-activit$" or fitbit or garmin or "pokemon go" or "apple health" or strava or "7 minute workout" or "30 day fitness" or "Wii-fit" or pedometer$ or runkeeper or mapmyfitness or myfitnesspal or acceleromet$ or IMU or "inertial measurement unit" or "couch to 5K").ti,ab,ot. (30001)

34 ((Activity or Fitness) adj3 challeng$).ti,ab,ot. (2036)

35 or/1-34 (1977842)

36 exp *adolescent/ (27919)

37 *young adult/ (2849)

38 *child/ or *boy/ or *girl/ or *school child/ (94247)

39 school/ or college/ or community college/ or high school/ or medical school/ or middle school/ or pharmacy school/ or primary school/ or university/ (362980)

40 ("Generation Z" or "Gen Z" or "Young person" or "young people" or "Younger generation" or "School age" or "School child" or "school children" or "young adult" or " young adults" or "School aged" or school or schools).ti,ab,ot. (503795)

41 (teen or teens or Teenager$ or schoolage$ or schoolchild$ or freshman or freshmen or sophomore$ or Student$ or pupil$ or adolescen$ or Millennial$ or "High school" or "Middle school").ti,ab,ot. (841236)

42 (College$ or Universit$ or Youth or youths or Boy$ or Girl$ or "Young man" or "Young men" or "Young woman" or "Young women").ti,ab,ot. (1368129)

43 or/36-42 (2377129)

44 exp *exercise/ (153513)

45 *Fitness/ (15163)

46 exp *walking/ (39793)

47 exp *physical activity/ (135661)

48 *sedentary lifestyle/ (4739)

49 *sedentary time/ (535)

50 *movement time/ (33)

51 exp *sport/ (76971)

52 *Bicycle/ (3073)

53 (Exercis$ or walk$ or bicycl$ or bike or bikes or run or running or jog or jogging or dance$ or dancing or aerobics or swim or swimming).ti,ab,ot. (809285)

54 (Hourly prompt$ or activity remind$ or physical education).ti,ab,ot. (6231)

55 (Playground$ or play-ground$ or playarea$ or play-area$ or Active$ play$).ti,ab,ot. (3462)

56 ("walking bus" or "walk to school" or "walk to work" or "daily mile" or "park and stride").ti,ab,ot. (223)

57 Sedentary.ti,ab,ot. (44315)

58 (Step adj3 (count$ or goal$ or target$)).ti,ab,ot. (6507)

59 (physical$ adj2 (inactiv$ or activ$)).ti,ab,ot. (182752)

60 or/44-59 (1064574)

61 35 and 43 and 60 (25015)

62 animal/ or animal experiment/ (4188253)

63 (rat or rats or mouse or mice or murine or rodent or rodents or hamster or hamsters or pig or pigs or porcine or rabbit or rabbits or animal or animals or dogs or dog or cats or cow or bovine or sheep or ovine or monkey or monkeys).ti,ab,ot,hw. (7016288)

64 or/62-63 (7016288)

65 exp human/ or human experiment/ (22445559)

66 64 not (64 and 65) (5343787)

67 61 not 66 (24670)

68 limit 67 to yr="2015 -Current" (12832)

69 exp *Prevention/ or prevent$.ti,ab,ot,hw. (2519074)

70 (Risk adj3 (reduc$ or lower$ or adjust$ or decrease$)).ti,ab,ot. (384819)

71 exp *health education/ (116183)

72 exp *health behavior/ (144447)

73 *Motivation/ (29049)

74 (promot$ or program$ or campaign$ or strateg$ or educat$ or literacy or attitude$ or polic$ or encourage$ or awareness or Mentoring or advertis$).ti,ab,ot. (4912803)

75 ((Health$ or chang$) adj3 (behaviour$ or behavior$)).ti,ab,ot. (153071)

76 ((Increas$ or improv$ or encourage$) adj2 (participat$ or motivat$)).ti,ab,ot. (18782)

77 or/69-76 (7099455)

78 68 and 77 (7986)

79 (letter or editorial or note).pt. (2732601)

**80 78 not 79 (7965)**

**Medline and In-Process, In-Data-Review & Other Non-Indexed Citations (Ovid): 2015-2021/06/21**

**Searched 22.6.21**

1 exp *Internet/ (46947)

2 *smartphone/ (3965)

3 *Wearable electronic devices/ (3120)

4 *Fitness trackers/ (473)

5 *Electronic mail/ (1388)

6 *Video recording/ (7782)

7 *Video game/ (4578)

8 *virtual reality therapy/ (626)

9 *virtual reality system/ (0)

10 *online social networking/ (172)

11 *social networking/ (2691)

12 exp *Computers, Handheld/ (6487)

13 *Mobile Applications/ (6183)

14 *telemedicine/is, mt (7967)

15 *Self-Help Devices/ (3735)

16 *Remote Sensing Technology/ (2064)

17 *Monitoring, Ambulatory/ (5591)

18 *Actigraphy/ (1542)

19 *WIRELESS TECHNOLOGY/ (2654)

20 (Internet or "World wide web" or app or apps or application or smartphone$ or phone$ or text messag$ or SMS or web-based or interactive or video or youtube or whatsapp or facebook or social media or Instagram or Email$ or Game$ or Gaming or subscription or twitter or tweet$ or snapchat).ti,ab,ot. (1206900)

21 (online adj3 (counsel$ or coach$ or diar$)).ti,ab,ot. (517)

22 ((remote$ or online or digital$) adj3 (deliver$ or element$ or program$ or schedul$ or counsel$ or advisor$ or group$ or participa$ or tracker$)).ti,ab,ot. (14825)

23 e-coach$.ti,ab,ot. (55)

24 ((digital$ or online$ or remote$ or tech) adj3 (application$ or solution$)).ti,ab,ot. (4621)

25 (smart watch$ or smartwatch$ or wearable$ or fittech or software or iphone or mobile device$ or vlog$ or vlogger$ or influencer$ or Phone track$ or i-phone or android).ti,ab,ot. (203092)

26 (Virtual reality adj3 (game$ or active$ or interface$ or system$ or simulat$ or device$ or display$ or exercis$)).ti,ab,ot. (3366)

27 (VR adj3 (game$ or active$ or interface$ or system$ or simulat$ or device$ or display$ or exercis$)).ti,ab,ot. (1760)

28 zoom.ti,ab,ot. (1897)

29 ((step or steps or fitness or activ$ or exercis$ or digital$) adj3 (count$ or device$ or monitor$ or app or apps or tracker$)).ti,ab,ot. (49706)

30 (Daily adj2 (step or steps or walk$)).ti,ab,ot. (2345)

31 (((remote$ or online or digital$ or "technology assisted") adj3 fitness) or tracker$).ti,ab,ot. (5120)

32 (activometer or "active-o-meter" or personal tracker$).ti,ab,ot. (0)

33 (Peloton or "e-fitness" or "e-activit$" or fitbit or garmin or "pokemon go" or "apple health" or strava or "7 minute workout" or "30 day fitness" or "Wii-fit" or pedometer$ or runkeeper or mapmyfitness or myfitnesspal or acceleromet$ or IMU or "inertial measurement unit" or "couch to 5K").ti,ab,ot. (22583)

34 ((Activity or Fitness) adj3 challeng$).ti,ab,ot. (1589)

35 or/1-34 (1486729)

36 exp *adolescent/ (5548)

37 *young adult/ (102)

38 exp *child/ or exp *student/ (94446)

39 *schools/ or *universities/ (35992)

40 ("Generation Z" or "Gen Z" or "Young person" or "young people" or "Younger generation" or "School age" or "School child" or "school children" or "young adult" or " young adults" or "School aged" or school or schools).ti,ab,ot. (390994)

41 (teen or teens or Teenager$ or schoolage$ or schoolchild$ or freshman or freshmen or sophomore$ or Student$ or pupil$ or adolescen$ or Millennial$ or "High school" or "Middle school").ti,ab,ot. (624784)

42 (College$ or Universit$ or Youth or youths or Boy$ or Girl$ or "Young man" or "Young men" or "Young woman" or "Young women").ti,ab,ot. (839975)

43 or/36-42 (1499087)

44 *Physical Fitness/ (17223)

45 *Cardiorespiratory Fitness/ (1559)

46 *Walking/ (20013)

47 *Walking speed/ (940)

48 *Stair climbing/ (150)

49 exp *exercise/ (142566)

50 exp *sports/ (133648)

51 *Sedentary Behavior/ (6001)

52 (Exercis$ or walk$ or bicycl$ or bike or bikes or run or running or jog or jogging or dance$ or dancing or aerobics or swim or swimming).ti,ab,ot. (591612)

53 (physical$ adj2 (inactiv$ or activ$)).ti,ab,ot. (130344)

54 (Playground$ or play-ground$ or playarea$ or play-area$ or Active$ play$ or gym or gyms).ti,ab,ot. (4442)

55 ("walking bus" or "walk to school" or "walk to work" or "daily mile" or "park and stride").ti,ab,ot. (168)

56 Sedentary.ti,ab,ot. (32719)

57 (Step adj3 (count$ or goal$ or target$)).ti,ab,ot. (4792)

58 (Hourly prompt$ or activity remind$ or physical education).ti,ab,ot. (4820)

59 or/44-58 (771385)

60 35 and 43 and 59 (15070)

61 exp animals/ not (exp animals/ and humans/) (4839846)

62 60 not 61 (14976)

63 limit 62 to yr="2015 -Current" (8116)

64 Prevent$.ti,ab,ot. (1482647)

65 (Risk adj3 (reduc$ or lower$ or adjust$ or decrease$)).ti,ab,ot. (257794)

66 exp *Health Education/ (148494)

67 exp *Health Behavior/ (176720)

68 exp *School Health Services/ or *motivation/ or *drive/ or *goals/ (52987)

69 (promot$ or program$ or campaign$ or strateg$ or educat$ or literacy or attitude$ or polic$ or encourage$ or awareness or Mentoring or advertis$).ti,ab,ot. (3757929)

70 ((Health$ or chang$) adj3 (behaviour$ or behavior$)).ti,ab,ot. (120126)

71 ((Increas$ or improv$ or encourage$) adj2 (participat$ or motivat$)).ti,ab,ot. (13230)

72 or/64-71 (5170428)

**73 63 and 72 (4976)**

**Medline Daily Update and Epubs Ahead-of-Print (Ovid): up to 2021/06/21**

**Searched 22.6.21**

1 exp *Internet/ (102)

2 *smartphone/ (34)

3 *Wearable electronic devices/ (31)

4 *Fitness trackers/ (5)

5 *Electronic mail/ (1)

6 *Video recording/ (18)

7 *Video game/ (16)

8 *virtual reality therapy/ (2)

9 *virtual reality system/ (0)

10 *online social networking/ (4)

11 *social networking/ (7)

12 exp *Computers, Handheld/ (37)

13 *Mobile Applications/ (60)

14 *telemedicine/is, mt (29)

15 *Self-Help Devices/ (3)

16 *Remote Sensing Technology/ (7)

17 *Monitoring, Ambulatory/ (7)

18 *Actigraphy/ (3)

19 *WIRELESS TECHNOLOGY/ (5)

20 (Internet or "World wide web" or app or apps or application or smartphone$ or phone$ or text messag$ or SMS or web-based or interactive or video or youtube or whatsapp or facebook or social media or Instagram or Email$ or Game$ or Gaming or subscription or twitter or tweet$ or snapchat).ti,ab,ot. (27431)

21 (online adj3 (counsel$ or coach$ or diar$)).ti,ab,ot. (36)

22 ((remote$ or online or digital$) adj3 (deliver$ or element$ or program$ or schedul$ or counsel$ or advisor$ or group$ or participa$ or tracker$)).ti,ab,ot. (925)

23 e-coach$.ti,ab,ot. (1)

24 ((digital$ or online$ or remote$ or tech) adj3 (application$ or solution$)).ti,ab,ot. (176)

25 (smart watch$ or smartwatch$ or wearable$ or fittech or software or iphone or mobile device$ or vlog$ or vlogger$ or influencer$ or Phone track$ or i-phone or android).ti,ab,ot. (5512)

26 (Virtual reality adj3 (game$ or active$ or interface$ or system$ or simulat$ or device$ or display$ or exercis$)).ti,ab,ot. (108)

27 (VR adj3 (game$ or active$ or interface$ or system$ or simulat$ or device$ or display$ or exercis$)).ti,ab,ot. (85)

28 zoom.ti,ab,ot. (97)

29 ((step or steps or fitness or activ$ or exercis$ or digital$) adj3 (count$ or device$ or monitor$ or app or apps or tracker$)).ti,ab,ot. (1021)

30 (Daily adj2 (step or steps or walk$)).ti,ab,ot. (80)

31 (((remote$ or online or digital$ or "technology assisted") adj3 fitness) or tracker$).ti,ab,ot. (217)

32 (activometer or "active-o-meter" or personal tracker$).ti,ab,ot. (0)

33 (Peloton or "e-fitness" or "e-activit$" or fitbit or garmin or "pokemon go" or "apple health" or strava or "7 minute workout" or "30 day fitness" or "Wii-fit" or pedometer$ or runkeeper or mapmyfitness or myfitnesspal or acceleromet$ or IMU or "inertial measurement unit" or "couch to 5K").ti,ab,ot. (638)

34 ((Activity or Fitness) adj3 challeng$).ti,ab,ot. (36)

35 or/1-34 (34148)

36 exp *adolescent/ (1)

37 *young adult/ (0)

38 exp *child/ or exp *student/ (208)

39 *schools/ or *universities/ (58)

40 ("Generation Z" or "Gen Z" or "Young person" or "young people" or "Younger generation" or "School age" or "School child" or "school children" or "young adult" or " young adults" or "School aged" or school or schools).ti,ab,ot. (9575)

41 (teen or teens or Teenager$ or schoolage$ or schoolchild$ or freshman or freshmen or sophomore$ or Student$ or pupil$ or adolescen$ or Millennial$ or "High school" or "Middle school").ti,ab,ot. (17402)

42 (College$ or Universit$ or Youth or youths or Boy$ or Girl$ or "Young man" or "Young men" or "Young woman" or "Young women").ti,ab,ot. (18598)

43 or/36-42 (34318)

44 *Physical Fitness/ (10)

45 *Cardiorespiratory Fitness/ (13)

46 *Walking/ (54)

47 *Walking speed/ (4)

48 *Stair climbing/ (1)

49 exp *exercise/ (332)

50 exp *sports/ (232)

51 *Sedentary Behavior/ (37)

52 (Exercis$ or walk$ or bicycl$ or bike or bikes or run or running or jog or jogging or dance$ or dancing or aerobics or swim or swimming).ti,ab,ot. (12343)

53 (physical$ adj2 (inactiv$ or activ$)).ti,ab,ot. (3746)

54 (Playground$ or play-ground$ or playarea$ or play-area$ or Active$ play$ or gym or gyms).ti,ab,ot. (99)

55 ("walking bus" or "walk to school" or "walk to work" or "daily mile" or "park and stride").ti,ab,ot. (7)

56 Sedentary.ti,ab,ot. (771)

57 (Step adj3 (count$ or goal$ or target$)).ti,ab,ot. (129)

58 (Hourly prompt$ or activity remind$ or physical education).ti,ab,ot. (92)

59 or/44-58 (15442)

60 35 and 43 and 59 (462)

61 exp animals/ not (exp animals/ and humans/) (6463)

62 60 not 61 (462)

63 Prevent$.ti,ab,ot. (29461)

64 (Risk adj3 (reduc$ or lower$ or adjust$ or decrease$)).ti,ab,ot. (7563)

65 exp *Health Education/ (133)

66 exp *Health Behavior/ (294)

67 exp *School Health Services/ or *motivation/ or *drive/ or *goals/ (62)

68 (promot$ or program$ or campaign$ or strateg$ or educat$ or literacy or attitude$ or polic$ or encourage$ or awareness or Mentoring or advertis$).ti,ab,ot. (90208)

69 ((Health$ or chang$) adj3 (behaviour$ or behavior$)).ti,ab,ot. (3645)

70 ((Increas$ or improv$ or encourage$) adj2 (participat$ or motivat$)).ti,ab,ot. (452)

71 or/63-70 (114283)

**72 62 and 71 (281)**
